# Integrated “omics” profiling indicates that miRNAs are modulators of the ontogenetic venom composition shift in the Central American rattlesnake, *Crotalus simus simus*

**DOI:** 10.1186/1471-2164-14-234

**Published:** 2013-04-10

**Authors:** Jordi Durban, Alicia Pérez, Libia Sanz, Aarón Gómez, Fabián Bonilla, Santos Rodríguez, Danilo Chacón, Mahmood Sasa, Yamileth Angulo, José M Gutiérrez, Juan J Calvete

**Affiliations:** 1 Instituto de Biomedicina de Valencia, Consejo Superior de Investigaciones Científicas, Jaime Roig 11, Valencia, 46010, Spain; 2 Instituto Clodomiro Picado, Facultad de Microbiología, Universidad de Costa Rica, San José, Costa Rica

**Keywords:** Ontogenetic venom shift, Venomics, Snake venom gland transcriptomics, 454 pyrosequencing, Ion-Torrent microRNA profiling, *Crotalus simus simus*

## Abstract

**Background:**

Understanding the processes that drive the evolution of snake venom is a topic of great research interest in molecular and evolutionary toxinology. Recent studies suggest that ontogenetic changes in venom composition are genetically controlled rather than environmentally induced. However, the molecular mechanisms underlying these changes remain elusive. Here we have explored the basis and level of regulation of the ontogenetic shift in the venom composition of the Central American rattlesnake, *Crotalus s. simus* using a combined proteomics and transcriptomics approach.

**Results:**

Proteomic analysis showed that the ontogenetic shift in the venom composition of *C. s. simus* is essentially characterized by a gradual reduction in the expression of serine proteinases and PLA_2_ molecules, particularly crotoxin, a β-neurotoxic heterodimeric PLA_2_, concominantly with an increment of PI and PIII metalloproteinases at age 9–18 months. Comparison of the transcriptional activity of the venom glands of neonate and adult *C. s. simus* specimens indicated that their transcriptomes exhibit indistinguisable toxin family profiles, suggesting that the elusive mechanism by which shared transcriptomes generate divergent venom phenotypes may operate post-transcriptionally. Specifically, miRNAs with frequency count of 1000 or greater exhibited an uneven distribution between the newborn and adult datasets. Of note, 590 copies of a miRNA targeting crotoxin B-subunit was exclusively found in the transcriptome of the adult snake, whereas 1185 copies of a miRNA complementary to a PIII-SVMP mRNA was uniquely present in the newborn dataset. These results support the view that age-dependent changes in the concentration of miRNA modulating the transition from a crotoxin-rich to a SVMP-rich venom from birth through adulhood can potentially explain what is observed in the proteomic analysis of the ontogenetic changes in the venom composition of *C. s. simus*.

**Conclusions:**

Existing snake venom toxins are the result of early recruitment events in the Toxicofera clade of reptiles by which ordinary genes were duplicated, and the new genes selectively expressed in the venom gland and amplified to multigene families with extensive neofunctionalization throughout the approximately 112–125 million years of ophidian evolution. Our findings support the view that understanding the phenotypic diversity of snake venoms requires a deep knowledge of the mechanisms regulating the transcriptional and translational activity of the venom gland. Our results suggest a functional role for miRNAs. The impact of specific miRNAs in the modulation of venom composition, and the integration of the mechanisms responsible for the generation of these miRNAs in the evolutionary landscape of the snake's venom gland, are further challenges for future research.

## Background

The presence of a venom system is a shared derived character of the advanced snakes [1-3]. Venom represents an adaptive trophic trait in snake evolution [4-7] that has played a central role in the origin of the advanced snakes during the Cenozoic era [8,9]. The diversity of modern snakes appeared during the Paleocene period of the Cenozoic Era, approximately 54–64 Mya, following the split of the Pareatidae from the remaining Caenophidians [6]. In Occidental culture, Francesco Redi (1626–1697), court physician to Ferdinando II de’ Medici, Grand Duke of Tuscany and his successor, Cosimo III, is credited for discovering how vipers produce venom and inject it into their prey [10]. One century latter, Abbé Gasparo Ferdinando Felice Fontana (1730–1805), first director of the Museum of Physics and Natural History in Florence, performed experiments on the venom of the European viper. His classic text published in 1781 [11] is regarded the Opera Prima of modern toxinology [12].

Research on venoms has been continuously advanced by technological developments [13]. Particularly for the past decade, and fueled by the application of "omic" technologies [14-18], the field of molecular toxinology has experienced a sustained exponential growth. In-depth venom proteomics and transcriptomic analyses have recently become available for a number of venomous snake lineages, revealing a vast unexplored source of evolutionary innovation [2,19,20]. Snake venoms are well documented as having different venom compositions and toxicity based on taxonomic or geographical locations [21]. Inter- and intraspecific individual, gender-specific, regional, and seasonal variations in venom toxin composition may reflect local adaptations conferring fitness advantages to individuals specializing on different prey, or phylogenetic carry on. Understanding the processes that drive the evolution of snake venom variability is a topic of intense research interest in molecular and evolutionary toxinology. Recent studies of the molecular basis of adaptations have sought to understand the relative importance of gene regulation effects as determinants of venom phenotype [6,22-24]. Most significantly, a number of snakes show age-related (ontogenetic) changes in venom composition [25-32]. Surprisingly, despite previous suggestions that ontogenetic changes in venom are prey-related [33], juvenile Dusky Pigmy rattlesnakes, *Sistrurus miliarius barbouri*, raised on different diets showed similar albeit highly-variable venom compositions by the end of a 26-months study, suggesting little effect of diet on the overall make-up of venom in snakes this age or younger [23]. Over the same period shifts in venom composition occurred in females raised on different prey in all diet treatments with the magnitude of those changes strongly related to diet. This work provided evidence that venom composition is somewhat plastic in both juvenile and adult *S. m. barbouri* and that, at least in adults, prey consumed may influence the relative abundance of possibly functionally-distinct classes of venom proteins [23]. However, the molecular mechanisms that underly age-related changes in venom remain elusive.

In this work we have explored the basis and level of regulation of the ontogenetic shift in the venom composition of the Central American rattlesnake, *Crotalus s. simus*. Biogeographical data indicate a basal cladogenesis in the Central American *C. simus* clade, dating back to the late Miocene/early Pliocene (6.4-6.7 Mya) [34]. Neonate and juvenile *C. s. simus* venoms contain large relative amounts of crotoxin, a heterodimeric PLA_2_ molecule exhibiting presynaptic β-neurotoxicity, along with low concentration of hemorrhagic snake venom metalloproteinases (SVMPs) [29]. By contrast, adult *C. s. simus* venom presents a high content of SVMP and is largely devoid of neurotoxic activity [29,35,36]. Juvenile and adult *C. s. simus* venom phenotypes broadly correspond, respectively, to type II (low metalloproteinase activity and high toxicity, LD_50_ <1 μg/g mouse body weight) and type I (high levels of SVMPs and low toxicity, LD_50_ >1 μg/g mouse body weight) venoms defined by Mackessy [37,38]. Here we investigate the transition from type II to type I venom phenotype in *C. s. simus* through an integrated "omics" approach involving proteomic survey of time-course venom composition variation, from birth through adulthood, and Next Generation sequencing of the venom gland transcriptomes and microRNAs of neonate and adult specimens. With this experimental design we intended to determine whether venom gene expression is transcriptionally controlled by developmental stage-dependent factors, or whether the ontogenetic changes in venom composition involve regulation at the post-transcriptional level.

## Results and discussion

### Time-resolved proteomic analysis of the ontogenetic changes in the venom composition of *C. s. simus*

A previous proteomic survey of the venom of the Central American rattlesnake, *C. s. simus*, laid the foundation for understanding the changes in toxin composition and overall pharmacological features between adult (predominantly hemorrhagic) and newborn (mainly neurotoxic) snakes [29]. This study revealed prominent stage-dependent protein expression changes between the pooled venoms from newborn and adult specimens from Costa Rica, characterized by a shift from a type II to a type I venom phenotype [29]. Such a conspicuous venom composition change has been hypothesized to be related to variations in the size of prey consumed by snakes of different ages and the variable requirements for immobilizing and digesting them [37,38]. It has also been suggested that the significance of venom adaptation to specific diets represents a trade-off between the metabolic cost of venom production and increasing foraging efficiency [7,39]. In the present work we have sought to establish the molecular mechanism responsible for the reported ontogenetic shift in venom protein composition.

**Figure 1:**
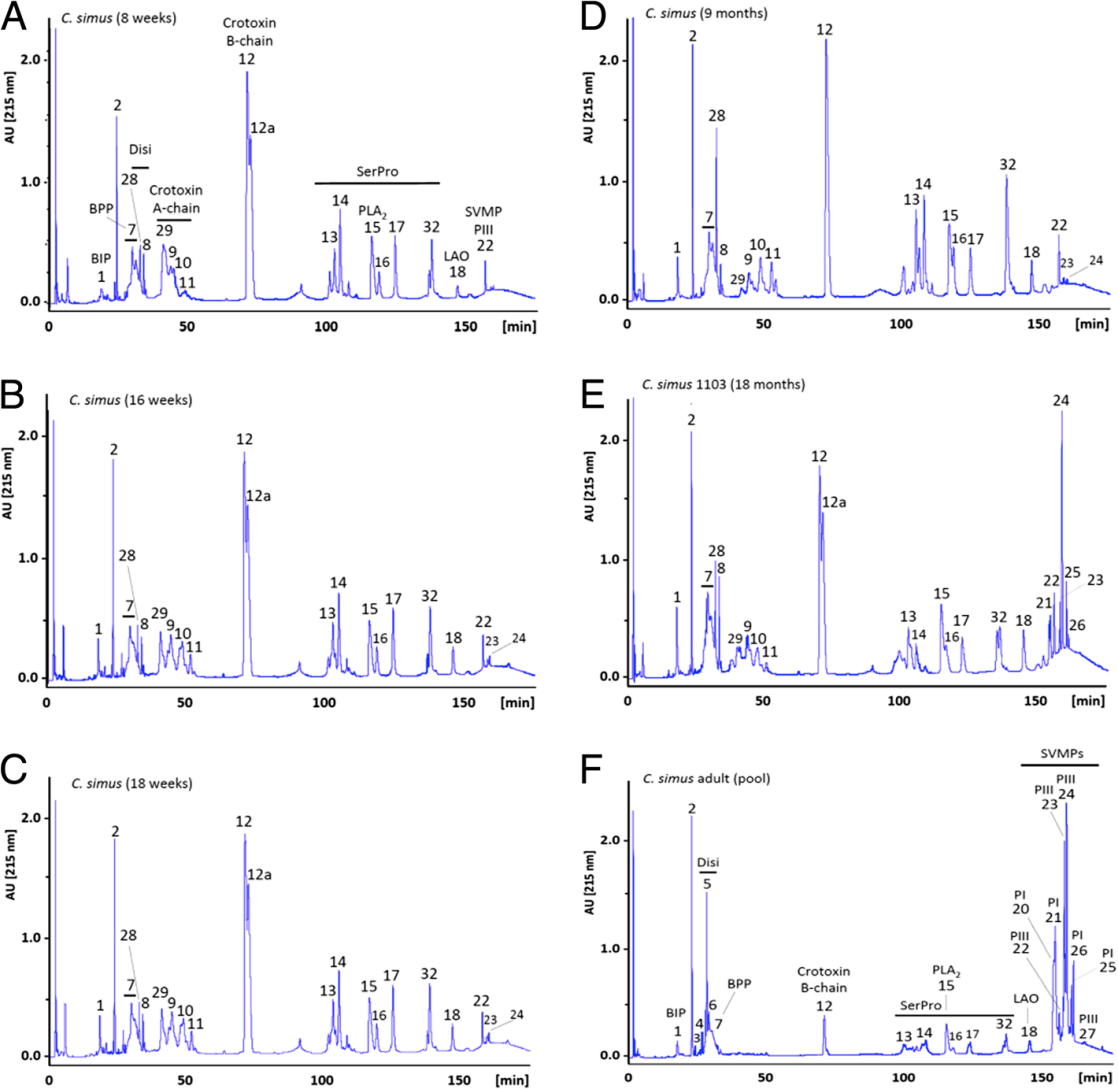
**Proteomic analysis of the ontogenetic changes in the venom composition of *C. simus*.** Panels **A**-**F** display reverse-phase HPLC profiles changes of pooled venoms collected from specimens from age 8-weeks to adulthood (≥36 months). Chromatographic fraction numbers and their identities correspond to those previously identified by Calvete *et al*. [Figure 1 and Table of ref. [29]. BIP, bradykinin-inhibitory peptide; BPP, bradykinin-potentiating peptides; Disi, disintegrin; SerProt, serine proteinase; PLA_2_, phospholipase A_2_; LAO, L-amino acid oxidase; PI and PIII, snake venom metalloproteases (SVMP) of class PI and PIII, respectively; Peaks 29, 9, 10, and 11, crotoxin A-chain [UniProtKB accession code P08878]; peak 12, crotoxin B-chain-1 [P62022]; peak 12a, crotoxin B-chain-2 [P24027].

**Figure 2:**
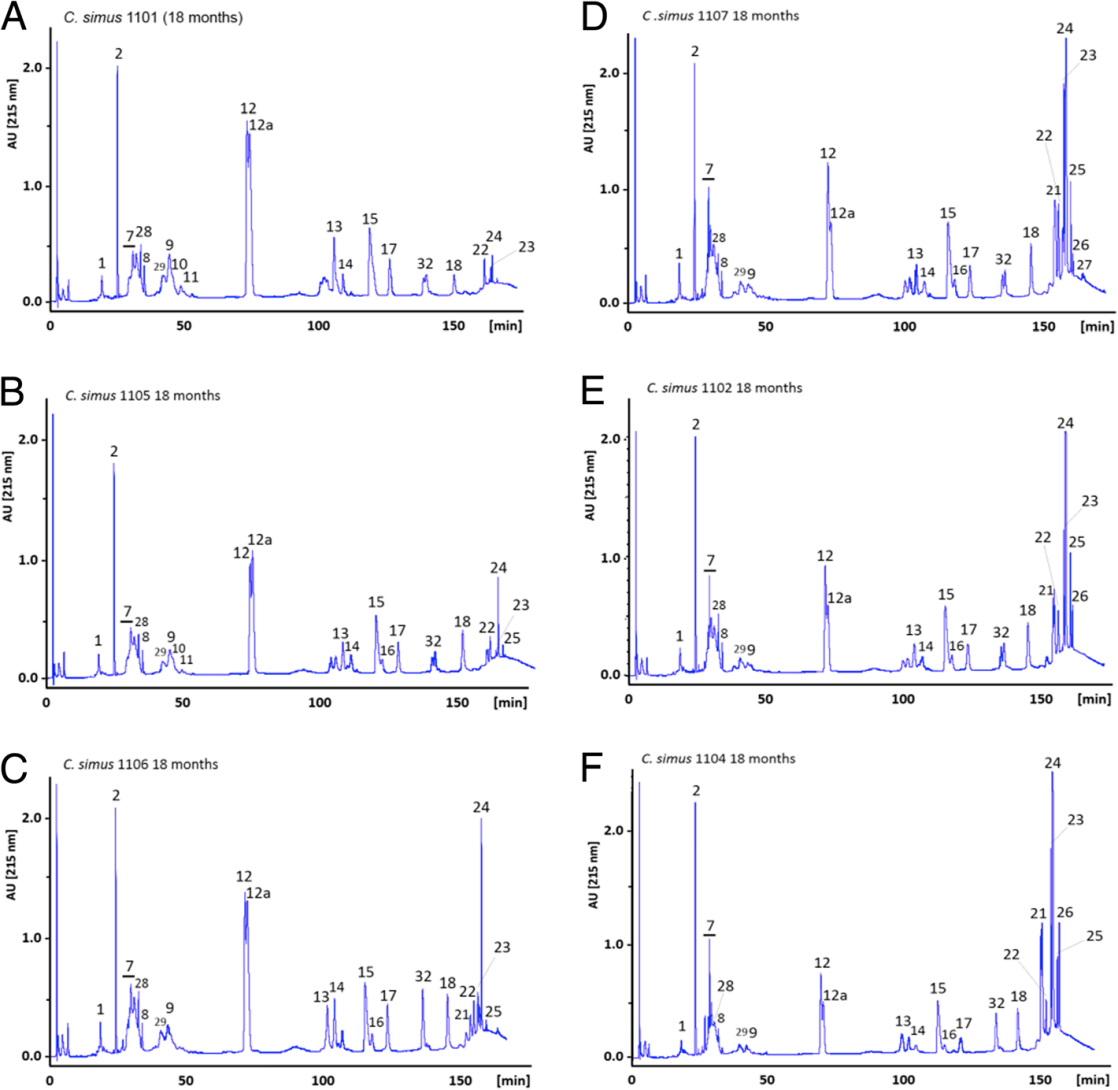
**Individual variability in the venom composition of 18-month old *C. simus***. Panels **A**-**F** display reverse-phase HPLC separations of the venom of six *C. simus* siblings illustrating the range of venom phenotypes observed at age 18 months. Peak numbering as in Figure 1.

Figure 1 presents reverse-phase HPLC profiles showing changes in the composition of venom pooled from specimens from age 8-weeks to adulthood (≥ 36 months). The ontogenetic shift is essentially characterized by a gradual reduction in the expression of serine proteinases and PLA_2_ molecules, particularly crotoxin, a β-neurotoxic heterodimeric PLA_2_[40-42], concomitantly with an increased secretion of PI and PIII SVMPs at age 9–18 months. Of particular note, whereas venoms from individual 9-month-old *C. s. simus* specimens showed indistinguishable reverse-phase chromatographic profiles, venoms from 18-month-old snakes exhibited large individual variation in their crotoxin and SVMP contents (Figure 2), suggesting a key point in shifts in venom composition at this developmental stage. Consistent with this view, juvenile specimens around this age exhibit a range of venom phenotypes, between predominantly type I, type II, and combinations of the two. Systemic effects involving hemostatic disturbances, i.e. coagulopathy, have not been documented in Central American rattlesnake envenomings [35,43]. However, there is little information on the clinical observations of envenomings induced by specimens of *C. simus* of various ages in Central America. Envenomings by adults are characterized by local tissue damage, i.e. edema and hemorrhage and systemic manifestations associated with cardiovascular effects and coagulopathy [43]. Our proteomic data suggest that bites by juvenile specimens might be characterized by a combination of SVMP-induced hemorrhage and crotoxin-induced neurotoxicy, in addition to serine proteinase-induce coagulopathy. Thus variable clinical manifestations might occur in accidents by *C. simus* of different ages. A similar trend regarding geographical differences in venom composition and toxicity has been described in the literature for the North American Mojave rattlesnake, *C. s. scutulatus*. Venom populations of *C. s. scutulatus* exhibit an intergradation pattern from SVMP-rich (type B) to Mojave toxin-rich (type A) phenotypes, from south central to southeastern Arizona [44]. Type A venom has large amounts of Mojave (crotoxin-like) toxin and shows neurotoxic effects. Type B venom has large amounts of SVMPs and shows hemorrhagic effects, and type A+B venom is a combination of the two and induces both neurotoxic and hemorrhagic effects [45,46]. Geographic venom variation throughout the *C. s. scutulatus* range correlated with clinical severity outcomes [44]. Hence, besides ecological and taxonomical implications, knowledge of the natural history and toxin composition of venoms is of fundamental importance for the treatment of bite victims and for the selection of specimens for the preparation of venom pools for antivenom production [47,48].

### Analysis of the venom gland transcriptomes of neonate and adult *C. s. simus*. Retroelements

To investigate the basis of the ontogenetic venom shift revealed by the time-resolved proteomic analysis, we compared the transcriptional activity of the venom glands of a neonate and an adult *C. s. simus* specimens, using 454 pyrosequencing and the bioinformatic processing strategy outlined in Durban *et al*. [15]. 408,505 and 349,170 raw 454 reads from adult and newborn venom gland transcriptomes, respectively, were quality trimmed using the Prinseq software and only reads having a Quality Value (QV) greater than 20 [49] (355,140 (adult) and 320,907 (newborn) Table 1) were considered for assembling with Newbler 2.6 software. The analysis yielded 33,408 (adult) and 24,136 (newborn) singletons, and among the resulting 6,484 (adult) and 6,047 (newborn) total contigs, 1.43% and 10.74% comprised only 2 reads. Table 1 displays a summary of the 454 sequencing statistics.

**Table 1:** 454 sequencing statistics

	**Total reads**	**Mean length read (nt)**	**Max. length read (nt)**	**Assembled reads (%)**	**Number of contigs**	**Mean length contig (nt)**	**Max. length contig (nt)**	**Singletons**
Newborn	320,907	567.5	1,193	276,729 (86.23)	6,047	560.87	5,857	24,136
Adult	355,140	535.4	1,320	296,470 (83.48)	6,484	777.88	5,947	33,408

Raw reads, Roche Signal Processing Application reads, and Newbler 2.6-assembled contigs were inspected for poly-N's, the presence of library construction tags, and their Quality Values (QV). Only reads showing a QV > 20 were assembled.

Contig sequences were inspected for repetitive elements using Repeat Masker. 97,204 bases (1.93% of adult *C. s. simus* venom gland transcriptome) and 65,742 nucleotides (1.94% of the newborn venom gland transcriptome) were masked with N characters, a large part of them comprising Short and Long INterspersed repetitive Elements (SINEs and LINEs) retroelements (Additional file 1: Tables S1 and S2). Transposable elements (TE) that propagate within the host genome via RNA intermediates occupy a large fraction of eukaryotic genomes. Their mobility and amplification represent a major source of genomic variation [50,51]. Retrotransposable elements have been reported in the transcriptomes of *Bothrops insularis* (4.1% of ESTs) [52], *Lachesis muta* (0.3%) [53], and *Philodryas olfersii* (4.1%) [54], in PLA_2_ genes from the venom gland of *Vipera ammodytes*[55,56] and *Protobothrops flavoviridis*[57,58], and in an *E. ocellatus* PIII-SVMP gene [59]. Although their functional relevance in the venom gland remains unknown, transposable elements appear to be overrepresented in UTRs of mRNAs of rapidly evolving genes [60], suggesting that they have played a role in the diversification and expansion of these gene families [61,62].

Sauria SINE have been characterized in all major lineages of squamate reptiles [62,63], and phylogenetic analysis of *E. ocellatus* Sauria SINEs [57] indicated that their origin correlates with the time frame of the evolution of the snake venom system. Sauria SINEs may have arisen by a fusion of a tRNA-related sequence with the 3' end of a LINE [64] more than 200 million years ago and are uniquely widespread in lepidosaurian genomes [62]. SINEs have no protein coding capacity, and their retrotransposition depends on a "target-primed reverse transcription" by autonomous partner LINEs, that encode an endonuclease for cleaving the genomic integration site and a reverse transcriptase to copy the RNA to DNA [65,66]. Since Sauria SINEs share an identical 3' sequence with Bov-B LINEs, it has been proposed [63] that they utilize *in trans* the enzymatic machinery of Bov-B LINEs for their mobility and dispersal throughout the genome. In squamates and turtles, CR1 and L2 LINES are also partners of diverse SINEs [63,67].

An overview of the landscape of retrotransposable elements reported in the sauropsida taxon, a sister group of mammals that includes all extant reptiles and birds, has recently emerged from analysis of the draft genomes of the red jungle fowl, *Gallus gallus*[68], and the green anole, *Anolis carolinensis*[69]. Whereas a single type of LINE, CR1, comprises over 80% of all interspersed repeats in the chicken genome (200,000 copies; 9% of the chicken genome), approximately 30% of the *A. carolinensis* genome is composed of a wide diversity of LINE and SINE mobile element families. Since SINEs are among the largest multigene families in reptilian genomes, they may act as sites for homologous recombination events between dispersed SINEs, resulting in a variety of genetic rearrangements, including duplication, deletion and translocation, that likely represent mechanisms that generates genetic diversity in reptilian genomes [70]. The prevalence of transposable elements in untranslated regions of mRNAs of recently expanded gene classes suggested that TEs could affect gene expression through the donation of transcriptional regulatory signals [60]. The indistinguishable distribution of TEs in the venom gland transcriptomes of neonate and adult *C. s. simus* (Additional file 1: Tables S1 and S2) argues against this type of transcriptional regulation to explain the ontogenetic shift in venom composition.

### Comparison of toxin-coding transcript distribution in newborn and adult *C. s. simus* transcriptomes provides clues for streamlining their divergent venom phenotypes

**Figure 3:**
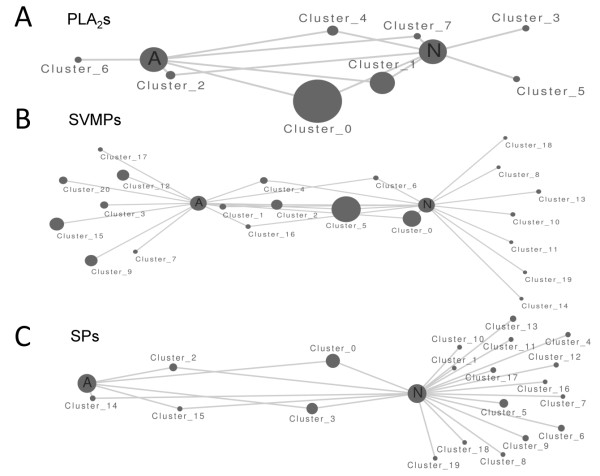
**Overview of shared and unique toxin clusters between adult and newborn *C. s. simus *transcriptomes.** Nodes represent protein clusters, with node size being proportional to read number in that cluster. Newborn (N) exclusive clusters are shown on the left, shared clusters in the middle, and adult (A) exclusive clusters on the right. Cluster distances to "A" and "N" are proportional to relative abundances in the transcriptome. Graphical view generated with Cytoscape (version 2.8.2) [71].

The sets of 6,484 (adult) and 6,047 (newborn) masked contigs were searched against the NCBI nucleotide sequence database using the BLASTN algorithm to identify similar sequences. 4,141 hits representing 63.9% of the total contigs of the adult snake transcriptome were retrieved, 431 of which (10.4% of matched hits) displayed similarity (e-value cutoff <10^-3^) to documented venom proteins of the taxonomic suborder *Serpentes*. In addition, 21,460 singleton sequences (64.23% of the total adult venom gland singletons, Table 1) produced significant BLASTN hits, and of these only 431 (2%) corresponded to documented snake venom entries.On the other hand, 3,022 (49.9% of the newborn venom gland transcriptome) found a BLAST hit, including 658 (10.9%) matches to snake venom proteins. Also, 526 sequences out of the 15,052 singleton BLAST hits (3.5%) corresponded to snake venom toxins. Additional file 2: Table S3 lists the relative abundances of the different venom protein family hits in the non-normalized venom gland transcriptomes of newborn and adult *C. s. simus*. The venom protein families identified in the newborn and adult venom gland transcriptomes, and the relative abundances of the length-normalized ORFs, simulated with NoiSeq for five technical replicates (nss=5), are displayed, are listed in Table 2. An estimation of the minimum number of genes from each toxin family transcribed into the venom gland transcriptome was calculated from the multiple alignments of reads matched to a full-length reference sequence [15] (Table 3). These newborn and adult synthetic gene sequences were clustered with CD-HIT into shared (identity threshold > 0.8) and unique clusters (Table 3). Figure 3 shows the distribution of clusters from the major toxin families between newborn and adult transcriptomes. These results indicated that both newborn and adult *C. s. simus* venom glands expressed non-overlapping gene sets. In particular, C-type lectin-like (CTL), (nerve growth factor) NGF, phospholipase A_2_ (PLA_2_), and snake venom metalloproteinase (SVMP) toxin families exhibited high NOISeq probabilities (*prob* value = 0.98, 0.93, 0.96, and 0.91, respectively) of being differentially expressed between the adult and the newborn transcriptomes. CTL, NGF, and SVMP toxin families were down-regulated in newborn *versus* adult database (-2.01, -1.69, and 0-77, respectively), whereas the PLA_2_ family appeared to be up-regulated (1.49).

**Table 2:** **RPKM (Reads per Kilobase per Million mapped reads)-normalized contigs and singletons from the venom gland transcriptomes of newborn and adult *C. s. simus *aligned to a reference snake venom toxin ORF**

	**Newborn**					**Adult**				
	**Contigs**	**Reads**	**Singletons**	**RPKM**	**%**	**Contigs**	**Reads**	**Singletons**	**RPKM**	**%**
5'-NTase	1	395	1	212.20	0.06	2	334	0	532.54	0.08
BPP	2	42	1	74.66	0.02	1	11	0	56.83	0.01
CRISP	3	62	0	81.26	0.02	0	0	0	0	0
CTL	6	483	5	1,035.42	0.31	13	1,462	4	9,255.5	1.5
GC	1	143	2	124.08	0.04	3	348	2	891.07	0.14
HYA	2	346	4	245.52	0.07	4	369	1	772.30	0.12
KUN	2	15	0	18.70	0.01	2	115	3	437.76	0.07
LAO	1	1,300	0	793.68	0.24	1	1,440	1	2,617.77	0.42
NGF	1	384	0	501.22	0.15	1	917	4	3,577.02	0.58
OHA	0	0	0	0	0	0	0	0	0	0
PDE	1	308	0	383.50	0.11	1	77	0	285.28	0.04
PLA_2_	27	41,700	37	94,942.21	28.37	18	11,015	9	74,618.46	12.13
SVMP	48	54,601	79	28,306.34	8.46	62	69,198	62	106,685.8	17.34
SP	191	156,291	194	207,689.54	62.07	83	105,104	62	415,323.2	67.51
VEGF	2	132	2	219.77	0.07	4	19	0	92.72	0.01

Abbreviations: *5'-NTase* 5'-nucleotidase, *BPP* bradykinin potentiating peptide, *CRISP* cysteine-rich secretory protein, *CTL* C-type lectin-like protein, *GC* glutaminyl cyclase, *HYA* hyaluronidase, *KUN* Kunitz-type inhibitor, *LAO* L-amino acid oxidase, *NGF* nerve growth factor, *OHA* ohanin, *PDE* phosphodiesterase, *PLA*_*2*_ phospholipase A_2_, *SVMP* snake venom metalloproteinase, *SP* serine proteinase, *VEGF* vascular endothelial growth factor. Protein families found in *C. s. simus* proteome [15] are underlined.

**Table 3:** **Estimation of minimum number of toxin genes transcribed into the venom gland transcriptomes of newborn (N) and adult (A) *C. s. simus***

	**Newborn**	**Adult**	**N + A**
**Toxin family**	**Unique**		**Shared**
5'-NTase			1
BPP	2	1	
CRISP	3		
CTL	6	2	
GC			1
HYA	1		1
KUN	2	2	
LAO			1
NGF			1
PDE			1
PLA_2_	2	1	5
SVMP	7	7	7
SP	15	0	50
VEGF	2	1	

Toxin family acronyms as in Table 2.

Protein families found in the respective venom proteomes [15] are underlined.

**Figure 4:**
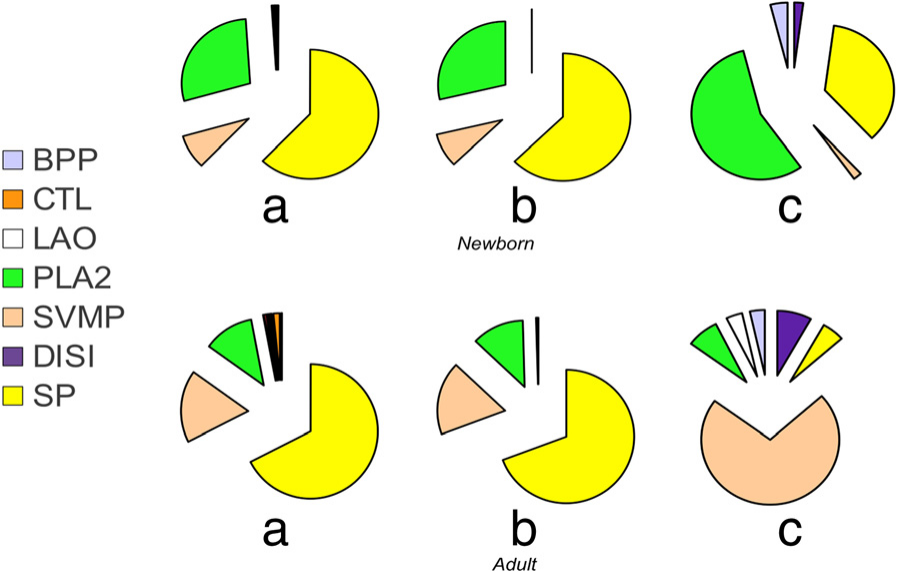
**Comparison of the protein composition of adult and newborn *C. s. simus *transcriptomes.** Chart pies “**a**” display the relative occurrence of RPKM-normalized ORF-coding reads listed in Table 2 in the venom gland transcriptomes of adult and newborn *C. s. simus*. Chart pies “**b**” display the same distribution as “**a**” but taking into account only protein families identified in the venom proteome [29]. Chart pies “**c**” show the toxin family composition of the venom proteomes of adult and newborn *C. s. simus* (data taken from [29]).

Figure 4 displays chart pies comparing the relative protein family compositions computed from the adult and newborn transcriptomes (Table 2) and the venom proteomes [15]. The amino acid sequences of transcript-deduced amino acid sequences of PLA_2_ molecules, serine proteinases, and SVMPs are shown in Additional file 3: Figures S1-S3). In line with previous transcriptomic surveys, the overall composition of neonate and adult transcriptomes (Figure 4, pie charts "a" and "b") are more similar to each other than their respective proteomes (Figure 4, pie charts "c"), indicating that the venom transcriptome may be subjected to stage-dependent translational control. In particular, newborn and adult venom glands expressed similar amounts of a transcript encoding a protein sequence 100% identical to crotoxin B-chain [P62022], whereas the concentration of this protein markedly differ in their respective venom proteomes (compare peak 12,12b in between panels of Figure 1). Moreover, shared clusters 1, 2, and 4 encode precursor crotoxin A-chain [P08878]-like sequences, although this protein (peaks 29, 9–11 in Figure 1), which is necessary for generating the heterodimeric presynaptic β-neurotoxic PLA_2_ molecule [40,41] responsible for the neurotoxicity of newborn and juvenile Central and adult South American rattlesnakes [42,43], is absent from the venom of adult *C. s. simus* venom [29]. Similarly, cluster 5, shared by newborn and adult venom gland transcriptomes (Figure 3B), encodes the PI-SVMP 20–21 exclusively found in venoms of juvenile (18-month) and adult specimens (Figure 1). Newborn and adult transcriptomes also share a number of clusters encoding PIII-SVMPs (0, 1, 2, 4, 6, and 16) although only a PIII-SVMP (peak 22, Figure 1) is present in the venom proteome of snakes aged 0–9 month. Moreover, PIII-SVMPs 8, 11, 14 and 18 and 9, 12, 15, 17 and 19 exhibited exclusive transcription in the newborn and the adult, respectively. On the other hand, although fragmentary, the protein sequences encoded by clusters 2, 12 and 15 match the MS/MS-derived peptide sequence information derived from the PIII-SVMPs 23–24 isolated from venom of adult snakes [29]. The higher diversity of serine proteinase transcripts characterized in the newborn *versus* the adult venom gland also mirrors the proteomic data (Figure 1). Shared cluster 0 (Figure 3C) encodes serine proteinase (SP) 14, an abundant enzyme in newborn and juvenile venom proteomes whose expression is significantly down-regulated in adult snakes (Figure 1). A precise matching of other venom serine proteinases characterized in *C. s. simus* is impeded by the lack of MS/MS data discriminating between the different transcript sequences (Additional file 3: Figure S3). Nevertheless, our results indicate that the age-dependent expression of both SP 14 and the major PIII-SVMPs found in adult *C. s. simus* venom might be due to a transcriptional regulatory mechanism. In addition, although it cannot be excluded that some shared transcripts correspond to pseudogenes, and also taking into consideration that the sampled transcriptomes were from single specimens, another main conclusion from our findings is that the molecular mechanism by which shared transcriptomes generate divergent venom phenotypes may operate post-transcriptionally.

### Distinct venom gland miRNA sets in newborn and adult snakes: modulators of the Central American rattlesnake's ontogenetic venom composition variation?

Phenotypic diversity generated through the regulation of RNA transcripts has been proposed as an explanation for the discrepancy between organismal complexity and the relatively limited number of primary coding transcripts [72,73]. Regulation by micro RNAs is one such mechanism [74,75]. MicroRNAs (miRNAs) are a class of small (~ 22 nucleotides in length), single-stranded, non-coding endogenous RNAs that are recently found to be negative post-transcriptional regulators of gene expression in eukaryotic organisms [76]. MicroRNAs act as adaptors that employ a silencing ribonucleoprotein complex to target mRNAs by selective Watson-Crick base-pairing, primarily in the 3'-UTR region. miRNAs anchored into specific binding pockets guide members of the Argonaute (Ago) protein family to target mRNA molecules for silencing or destruction [77]. The evolutionary dynamics of miRNAs across metazoan phylogeny and through deep evolutionary time suggests that metazoan morphological complexity might have its roots in miRNA innovation [78]. To explore the possible involvement of miRNAs in the post-transcriptional regulation of *C. s. simus* venom gland transcriptome, miRNA libraries from neonate and adult specimens were sequenced on an Ion Torrent Personal Genome Machine. Table 4A displays a summary of candidate miRNA sequencing statistics. Candidate miRNAs were filtered out according to nucleotide length and the presence of either the 30-mer IonA or the 23-mer P1 adapter sequences. This quality processing step yielded 38,738 (newborn) and 64,493 (adult) clusters, of which, respectively, 238 and 419 comprised at least 100 reads (Table 4B). These newborn and adult datasets contained 132 and 268 unique miRNAs, respectively, and 151 were shared between them (Table 4B). Although to date no snake miRNA has been reported in miRBase (http://www.mirbase.org), which includes 21,643 mature miRNA products from 168 species [79], BLAST analysis of *C. s. simus* the adult and newborn miRNA datasets against the mature miRBase retrieved 118 hits matching such diverse taxa as mammals (gray short-tailed opossum*, Mono-delphis domestica;* platypus*, Ornithorhynchus anatinus;* Tasmanian devil, *Sarcophilus harrisii*; bull, *Bos taurus;* horse, *Equus caballus*; Sumatran orangutan*, Pongo abelii*; wild boar, *Sus scrofa*; mouse, *Mus musculus,*), birds (zebra finch, *Taeniopygia guttata*)*,* fishes (sea lamprey, *Petro-myzon marinus*; Japanese ricefish, *Oryzias latipes*; zebra fish, D*anio rerio*), reptile (Anole lizard, *Anolis caro-linensis*), and the solitary sea squirt, *Ciona intestinalis* (Ascidian). However, 52 miRNA sequences (44%) cor-responded to miRNAs reported in *Anolis carolinensis*, the only available squamate genome [69].

**Table 4:** A. Summary of candidate miRNA sequencing statistics. B. Clustering and miRBase annotation statistics

**A**	**Total raw reads**	**Prinseq quality filtered reads**	**FastX too-short reads**	**FastX adapter-only reads**		**Processed reads**	**Mean length read (nt)**
Newborn	314,592	263,517	34,579	8,390		220,548	24.58
Adult	515,040	450,902	55,882	5,956		389,064	23.68
**B**	**Total clusters**	**≥100 reads**	**Unique clusters**	**Shared clusters**	**BLAST miRBase hits**	**Anolis hits**	**% Anolis hits**
Newborn	38,738	283	132	151	48	22	45.8
Adult	64,493	419	268	151	70	30	42.8

Total clusters were obtained after the CD-HIT usage. Clusters were compared between newborn and adult datasets and blasted against miRBase.

**Figure 5:**
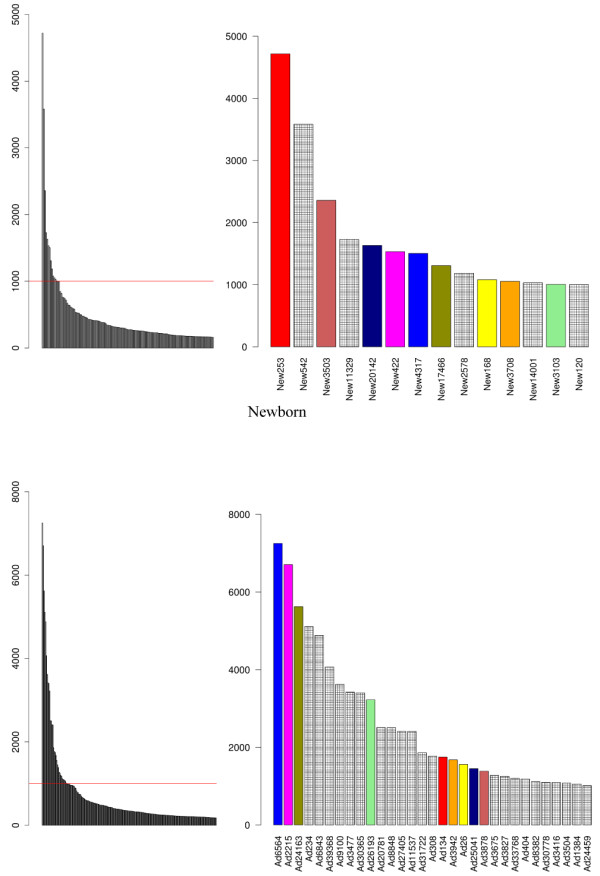
**Distribution of miRNAs in newborn and adult venom gland transcriptomes.** Left, uneven distribution of the number of miRNAs identified in the venom gland transcriptomes of newborn (upper panel) and adult (lower panel). The red line marks the 1000 copy threshold. Right, Comparison of highly expressed (>1000 counts) venom gland miRNAs in newborn (upper panel) and adult (lower panel) *C. s. simus* datasets. Bars corresponding to miRNAs shared between newborn and adult transcriptomes are filled with the same color. For details of the expression profile of highly expressed miRNAs in the venom gland transcriptome, and their newborn to adult expression ratios, please consult Tables S4 and S5 (Additional File 1).

**Figure 6:**
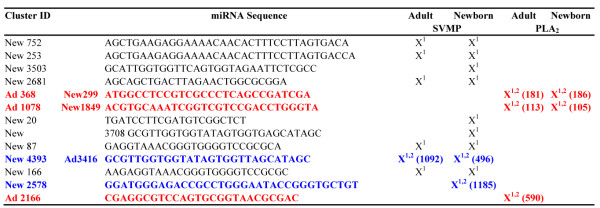
**miRNA sequences predicted by miRanda (1) and MapMi (2) to target 3'-UTR loci of SVMP (blue) and PLA2 (red) transcripts.** miRNA counts in their respective transcriptome are indicated in parentheses. Best BLAST hits of the clusters mapped by the miRanda- and MapMi-predicted miRNAs are listed in Figure 7. Best BLAST hits for miRanda-only-predicted miRNA sequences are displayed in Additional file 2: Table S6.

**Figure 7:**
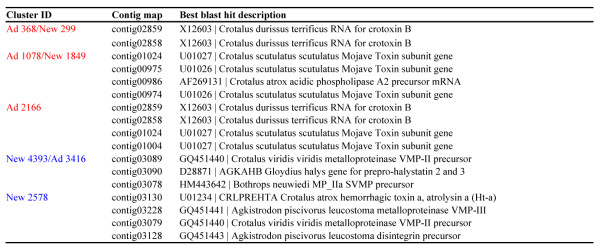
**Best BLAST hit descriptions of the 454 contigs mapped by the miRNA sequences highlighted in Figure**6**.** Predicted MapMi-calculated Watson-Crick pairing and binding energy to their target 3'-UTR loci are displayed in Figure S4 (Additional file 3).

MicroRNAs with frequency count of 1000 or greater exhibited a distinct distribution between the newborn and adult datasets (Figure 5). Noteworthy, the most expressed miRNAs in the newborn venom gland showed a significantly lower abundance in adults and *visa versa*, miRNAs abundantly expressed in adults have a lower expression in neonates (Figure 5, right panels; Tables S4 and S5 in Additional file 2). This uneven distribution of miRNAs suggests a regulatory mechanism by which a single transcriptome may result in different proteomes. Prediction of putative target genes was assessed by miRanda using the parameter specified in the Methods section. MiRanda is a bioinformatic tool for finding genomic targets for microRNAs that incorporates current biological knowledge on target rules and computes optimal sequence complementarity between a set of mature microRNAs and a given mRNA using a weighted dynamic algorithm [80-82]. MiRanda predicted 10 miRNAs complementary of 3'-UTR loci of *C. s. simus* SVMP 454-transcripts (5 shared between newborn and adult; 5 newborn-exclusive) and 3 miRNAs from adult snakes targeting PLA_2_ mRNAs (Figure 6; but see also Figure S4 in Additional file 3). When these matches were filtered through MapMi using thermodynamics, sequence conservation, and pairwise alignment criteria [83], positive hits were restricted to the three miRNA sequences mapping to PLA_2_ loci (clusters New299/Ad368, New1849/Ad1078, and Ad2166) and a pair of miRNAs against SVMP mRNAs (clusters New4393/Ad3416 and New2578) (Figure 6). miRNA counts in the newborn and adult venom gland transcriptomes, and best BLAST hits of their putative target genes are displayed in Figure 7. In addition, Table S6 (Additional file 2) lists the miRanda-only predicted miRNAs and their best BLAST hit, and Figure S4 (Additional file 3 shows their Watson-Crick pairing to target 3'-UTR loci of PLA2 and SVMP 454 transcripts, and the corresponding binding energy calculated by MapMi. Noteworthy, 590 copies of miRNA 2166, targeting crotoxin B-subunit, was exclusively found in the transcriptome of the adult snake whereas 1185 copies of miRNA 2578, complementary to a PIII-SVMP mRNA, was uniquely present in the newborn dataset (Figure 6).

**Figure 8:**
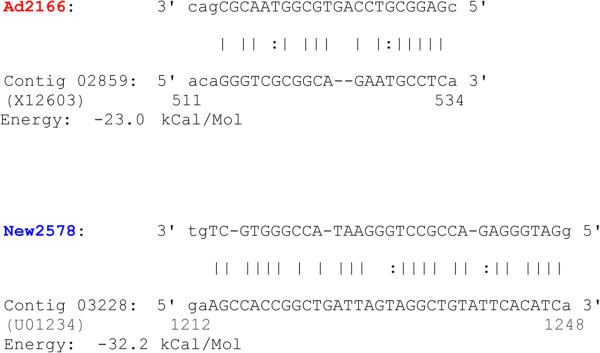
**Predicted miRNA binding features.** miRanda alignment of miRNAs from adult (Ad) and newborn (New) venom gland transcriptomes and their predicted target mRNA loci listed in Figure 7, showing their Watson-Crick pairing to 3'-UTR loci of PLA_2_ and SVMP 454 transcripts, and the MapMi-calculated binding energies. The adult-specific miRNA targeting crotoxin B-subunit is highlight in red; the miRNA selectively found in the newborn dataset and targeting a SVMP mRNA is colored blue. Nucleotide positions within the best BLAST hit in the NCBI database are indicated.

Animal miRNAs guide proteins to repress the translation of target mRNAs via imperfect base pairing between the miRNA and the target [75]. Although the precise rules for pairing between a miRNA and its mRNA target sites are not known, an obvious requirement for miRNA regulation is the concurrent expression of both a miRNA and its target genes, and requiring conserved Watson-Crick pairing to the 5' region of the miRNA centered on nucleotides 2–7 (the so-called miRNA "seed") markedly reduces the occurrence of false-positive predictions [84,85]. However, a modest role for 3'-supplementary in targeting specificity, and the rare occurrence of 3'-compensatory sites that can compensate for a single-nucleotide bulge or mismatch in the seed region, both centered on miRNA nucleotides 13-16/17, have been reported [86]. In addition, mismatch at position 1 is supported by biochemical and crystallographic studies, indicating that the 5'-most nucleotide of an Argonaute-bound guide RNA is not paired to the target strand [87,88]. Figure 8 and S6 (Additional file 2) display the complementarity between the dataset-exclusive miRNAs and their (miRanda + MapMi)-predicted PLA_2_ and SVMP target mRNA loci listed in Figure 7.

## Concluding remarks and perspectives

The most important concept that emerges from our results is the possibility that miRNAs play a role in both the ontogenetic shift observed in certain venoms and in the plasticity of venoms from adult snakes underlying adaptations to changing environments. An important caveat of our current understanding of miRNA target recognition and post-transcriptional venom gland transcriptome regulation is the absence of mapeable snake genomes. Nonetheless, almost half of the most abundant miRNAs sequenced from the venom gland of both, newborn and adult, *C. s. simus* were orthologous to sequences in *A. carolinensis*. More significant is the fact that a *C. s. simus* newborn venom gland-exclusive miRNA and a venom gland miRNA uniquely found in adult snakes target, respectively, mRNAs encoding a PIII-SVMP and the B-subunit of crotoxin/Mojave toxin. Relevant to this point, Mojave toxin is a neurotoxic heterodimeric PLA_2_, whose translation into the venom proteome is ontogenetically regulated [29] (Figure 1). The noncovalent association of two dissimilar subunits, the small acidic, nonenzymatic, and nontoxic A-subunit with the basic and weakly toxic PLA_2_ B-subunit increases the lethal potency of the uncomplexed crotoxin B-subunit by enabling the toxin to reach its specific site of action at the neuromuscular junction [40,41,89-91]. Hence, miRNAs targeting a crotoxin subunit messenger would serve the goal of eliminating the generation of the neurotoxic heterodimer. Age-dependent changes in the concentration of miRNA modulating the transition from a crotoxin-rich to a SVMP-rich venom from birth through adulthood can potentially explain what is observed in the age-dependent proteomic analysis of the ontogenetic changes in the venom composition of *C. s. simus* illustrated in Figure 1.

Large-scale proteomic analysis, performed for one miRNA (miR-223) in only one cell type (murine neutrophils), revealed that although some proteins were repressed by 50-80%, miR-223 typically had more modest effects [92], suggesting that perhaps other miRNAs in their endogenous context have targets for which protein output is more dramatically repressed. Clearly, a challenge ahead in molecular toxinology is to design laboratory experiments to uncover the impact, molecular details, and evolution of the regulatory miRNA-target interactions that shape venom phenotype during snake development. In this regard, it has been proposed that a single origin of venom in Squamata, the order of reptiles including lizards and snakes, dates back roughly 200 Mya to the Late Triassic/Early Jurassic period [1,2,93]. Existing snake venom toxins are the result of recruitment events by which ordinary genes were duplicated, and the new genes selectively expressed in the venom gland and amplified to multigene families with extensive neofunctionalization throughout the approximately 112–125 Mya of snake evolution. Given the central role that diet has played in the adaptive radiation of snakes [4], venom thus represents a key adaptation that has played an important role in the diversification of snakes. Our findings here reported support the view that understanding the basis for the phenotypic diversity of snake venoms requires a deep understanding of the mechanisms regulating the transcriptional and translational activity of the venom gland. Our results, though restricted to individual specimens from a single species, suggest a functional role for miRNAs. The impact of specific miRNAs in the modulation of venom composition, and the integration of the mechanisms responsible for the generation and impact of these miRNAs on patterns of expression in the snake's venom gland, are further challenges for future research.

## Methods

### Proteomics assessment of the ontogenetic shift of venom composition in C. simus

Venoms from a large number (>25) of adult *Crotalus s. simus* specimens and from 11 captive-born siblings (from an age of 8-weeks to 21-months) kept at the serpentarium of the Instituto Clodomiro Picado, University of Costa Rica in San José were collected by snake biting on a parafilm-wrapped jar (adults and juveniles) or by aspiration from the fangs of neonates using an Eppendorf pipette. Crude venoms were centrifuged at low speed to remove cells and debris, lyophilized, weighed on a microbalance, and stored at −20°C until used. Venom proteins were separated by reverse-phase HPLC using a Teknokroma Europa C_18_ (0.4 cm × 25 cm, 5 mm particle size, 300 Å pore size) column and an Agilent LC 1100 High Pressure Gradient System equipped with DAD detector and micro-Auto-sampler. The flow-rate was set to 1 ml/min and the column was developed with a linear gradient of 0.1% TFA in water (solution A) and acetonitrile (solution B), isocratically (5% B) for 10 min, followed by 5–25 % B for 20 min, 25-45% B for 120 min, and 45-70% for 40 min. Isolated proteins were characterized as described [29].

### Snake venom gland cDNA synthesis and 454 sequencing

Venom glands of an 8-week-old and an adult *C. s. simus* specimens were removed 3 days after venom milking, when transcription is maximal [94], from anesthetized snakes using fine forceps and immediately placed in RNAlater™ solution (Qiagen). About 50 mg of tissue were disrupted and homogenized by a rotor-stator homogenizer, and total RNA was isolated using RNeasy Mini kit (Qiagen), quantified in a NanoDrop ND_spectrophotometer (NanoDrop Technologies, Wilmington, DE, USA) through the A260/A280 ratio, and quality-checked on an agarose gel discerning the 28S and 18S bands of ribosomal RNA. First strand cDNA was synthesized using RevertAid™ H Minus First Strand cDNA Synthesis Kit (Fermentas), which selectively transcribes full-length polyadenylated mRNA. The manufacturer's recommendations were followed except where specified. Approximately 5 μg of total RNA was used as starting material. In order to avoid polymerase slippage, a modified 3' 54-mer adaptor (5' GAGCTAGTTCTGGAG(T)_16_VN), which includes a type IIs enzyme (GsuI) site (underlined), was used for first-strand synthesis. This modified oligonucleotide effectively converts the long run of adenosine residues at the polyA tail into a sequence that causes fewer problems for dideoxy sequencing chemistry, and thus the resulting cDNA libraries were enriched in 3'-end-transcripts. To avoid internal cuts, the cDNA was hemimethylated by adding 5-methyl-dCTP to the dNTPs mix. The first strand cDNA was used as template for second strand synthesis by *E. coli* DNA Polymerase I and RNase H. Double strand (ds) cDNA was precipitated with ethanol and the pellet was resuspended in 70 μL of nuclease-free water and subjected to enzymatic digestion with GsuI for 4 hours at 30°C. The enzyme was then inactivated at 65°C for 20 minutes and the digested cDNA was stored at −20°C. For 454 pyrosequencing, the GS FLX General DNA Library Preparation Method Manual workflow (Roche Diagnostics) was followed. To this end, 3 μg of final non-normalized cDNA library were sheared by nebulization into small fragments. The fragment ends were polished and short A/B adaptors were ligated onto both ends, providing priming regions to support both emulsion amplification and the pyrosequencing process. A biotin tag on the B adaptor allowed immobilization of the dscDNA library fragments onto streptavidin-conjugated magnetic beads and the subsequent isolation of the library of single strand cDNA sequencing templates. Each of the eight cDNA libraries was tagged with a unique 10-base sequence (MID, Multiplex IDentifier) that is recognized by the sequencing analysis software, allowing for automated sorting of MID-containing reads. Barcoded libraries were simultaneously sequenced in a Genome sequencing FLX Titanium System (Roche Applied Science) at Life Sequencing S.L. (Parc Científic Universitat de Valencia, Paterna, Valencia, Spain; http://www.lifesequencing.com) using the method developed by Margulies et al. [95].

### Bioinformatic analysis of the 454 reads

454 data were analyzed using the workflow developed in [15] to identify sequences of toxin molecules by similarity search against nucleotide databases, which includes available NGS algorithms and in-house scripts. An initial quality test step of both the raw reads provided by the Genome Sequencer FLX System prior to the assembly process and the longer contig sequences obtained after running the 454 Newbler assembler program (version 2.6) (Titanium chemistry) was run using the Prinseq program (standalone version 0.17.4) [96]. Interspersed repeats and low complexity DNA sequences were masked from the transcript reads using RepeatMasker (version 3.2.9) [97]. RepeatMasker is available from the Institute for Systems Biology (http://www.systemsbiology.org) addressing http://repeatmasker.org. The program screens DNA sequences for interspersed repeats and low complexity DNA sequences included in the Repbase database (http://www.girinst.org). Repbase, a comprehensive database of repetitive element consensus sequences (update of 20 September 2011), operates as a service of the Genetic Information Research Institute (http://www.girinst.org). Data and computational resources for the Pre-Masked Genomes page is provided courtesy of the UCSC Genome Bioinformatics group (http://genome.ucsc.edu). Masked contig sequences were translated into the 6 possible reading frames and blasted against the non-redundant NCBI database (http://blast.ncbi.nlm.nih.gov, release of February 2012) and the UniProtKB/Swiss-Prot Toxin Annotation Program database (http://us.expasy.org/sprot/tox-prot), using BlastX and BlastN [98] algorithms (version 2.2.24), specifying a cut-off value of e-03 and BLOSUM62 as scoring matrix. Snake venom gland-specific transcripts were selected from best BLAST-hit descriptions identifying GenBank entries belonging to the taxonomic suborder *Serpentes*. This taxonomic group is represented by 44,141 nucleotide records comprising entries from 2,396 different species. A second filtering round was carried out using a list of keywords (including the acronyms of all known toxin protein families described so far to distinguish putative snake venom toxins from non-toxin (ribosomal, mitochondrial, nuclear, etc.) ordinary proteins [15]. The phylogenetically nearest top-hit full-length sequence was designated as the reference sequence onto which all toxin family-specific reads were aligned to create a multiple alignment using COBALT [99]. The multiple alignment was then parsed to create an assembled (consensus) toxin sequence in which each amino acid position is supported by at least four reads. We considered two contigs as different if their nucleotide sequences depart in more number of positions than expected from a sequencing error rate of 1.5%, and the same mutated residues were found in at least two other reads. Positions where two or more amino-acids fulfilled this criterium were annotated as variable residues suggesting the occurrence of different alleles (isoforms) of the protein.

The relative expression of a given toxin protein family was calculated according to the RPKM (Reads per Kilobase of exon per Million mapped reads) standard procedure described by Mortazavi and coworkers [100]. This normalization procedure provides an analog of the relative molar concentrations of transcripts. To this end, all the reads contributing to the contigs (regardless whether that read uniquely maps to that contig or not) and the length of the phylogenetically nearest coding sequence were taken into account to calculate the RPKM normalized figure:

$$\mathrm{RPKM}=\begin{array}{c} \underline{\mathrm{ORF}\;\text{reads}+\mathrm{ORF}\;\text{Singletons}}\\ \begin{array}{ccc} \underline{\text{Total}\;\text{reads}} & \times & \underline{\text{Length}} \end{array}\\ \begin{array}{ccc} 10^{6} & & \;10^{3} \end{array} \end{array}$$

Possible differential expression of venom proteins between adult and the newborn individuals was assessed with the non-parametric NOISeq-sim algorithm [101] using the following parameters recommended for counts without replicates in the NOISeq-sim manual were used: k (counts equal to zero) = 0.5; nss (number of samples to be simulated) ≥ 5; pnr (percentage of the total sequencing depth) = 0.2; v (variability in the total sequencing depth of the simulated sample) = 0.02. Reads were normalized by the length of the phylogenetically nearest sequence and a threshold of 0.9 was allowed.

### Comparison of newborn and adult *C. s. simus* venom transcriptomes

To assess the degree of similarity between transcripts synthesized by newborn an adult venom glands, a Perl script was written that aligned singletons and Newbler-assembled contigs onto the open reading frame (ORF) of the phylogenetically nearest protein sequence used as reference. The aligned nucleotide sequences were re-assembled with MIRA (http://www.chevreux.org/projects_mira.html) to infer the minimum number of different assemblies. These resulting sequences from newborn and adult individuals were compiled into a single FASTA file, translated into protein sequences, and manually inspected to discard possible mispaired BLAST annotations of local regions due to incorrect frame translations. Protein sequences were then clustered with CD-HIT (standalone version 4.5.7 built on 27th February 2012) [102,103] to identify protein sequences shared between newborn and adult transcriptomes.

### Snake venom gland microRNA profiling

Total RNA from neonate and adult venom glands was used to isolate small RNA libraries using a RNeasy Mini Kit following the manufacture's (Qiagen) instructions. Samples were quantified using a NanoDrop ND-1000 spectrophotometer (NanoDrop Technologies, Wilmington, DE, USA) through the 260/280 absorbance relation. 5 microliter of neonate (258.6 ng/microliter) and adult (490.7 ng/microliter) size (<200 nt)-enriched microRNA (miRNA) library were sequenced on an Ion Torrent Personal Genome Machine (PGM™) Sequencing platform at Life Sequencing S.L.

### Bioinformatic processing of the Ion-Torrent miRNA reads

Using Prinseq [96], the Ion-Torrent miRNA reads were filtered out according to nucleotide length (min_length 15 nt; max_length 40 nt) and the presence of either the 30-mer IonA or the 23-mer P1 adapter sequences. Adapter sequences were removed using the fasxt_clipper tool from the Fastx-Toolkit (http://hannonlab.cshl.edu/fastx_toolkit). The resulting reads were clustered using CD-HIT-454 [102] setting an identity threshold of 0.98 and the accurate mode option g1. HIT-454 was designed to identify artificial duplicates from raw 454 sequencing reads, including exact duplicates and near identical duplicates. Script cdhit-cluster-consensus (v.13) of the CD-HIT suite of programs was run to derive consensus sequences for each of the miRNA clusters. This program is currently maintained by Dr. Li's group (http://weizhong-lab.ucsd.edu). The relative abundances of miRNAs with frequency count ≥ 100 were normalized by scaling the number of reads clustered by CD-HIT-454 to the total number of processed reads (220,548 for newborn + 389,064 for adult). To identify unique and shared sequences between the newborn and adult datasets, these newborn and adult miRNA clusters were compared between themselves using CD-HIT. Differential expression between miRNAs in these two datasets was assessed with NOISeq-sim [101] allowing a threshold of 0.9. miRNAs comprising ≥ 100 reads were subjected to BLAST analysis against miRBase (release 18, November 2011, which included 21643 mature miRNA products from 168 species) (http://www.mirbase.org) [79].

### The search for miRNA targets

The precise rules and energetics for pairing between a miRNA and its mRNA target sites are not completely understood [104,105]. A variety of computational algorithms aimed at predicting miRNA target genes incorporate rules for miRNA-mRNA interactions such as base pairing complementarity and favourable miRNA-mRNA duplex thermodynamics. Current prediction methods are diverse, both in approach and performance [106]. We have employed the freely available target prediction, position-weighted local alignment miRanda algorithm (standalone version 3.3a) [80,81] and the MapMi webserver (version 1.5.0-build 01, release March 2012) (http://www.ebi.ac.uk/enright-srv/MapMi) [83] to identify candidate miRNA target sites in 3'-UTR regions of 454 reads by base complementarity, and putative miRNA loci, respectively. MapMi is a tool designed to locate miRNA precursor sequences in existing genomic sequences, using potential mature miRNA sequences as input. miRanda is an algorithm for finding genomic targets for microRNAs that incorporates current biological knowledge on target rules and computes optimal sequence complementarity between a set of mature microRNAs and a given mRNA using a weighted dynamic programming algorithm. In addition, miRanda uses an estimate of the free energy of formation of the miRNA:mRNA duplex as a secondary filter [82]. The following parameters were set to reduce the estimated false positives to ≤ 9% [80,81]: total Score >100; ΔG of the intermediate duplex < −19 Kcal/mol; and output of 2 or more 454-contig targets.

### Database accession

The raw 454 GS FLX Titanium reads of *C. s. simus* adult and neonate venom gland transcriptomes, and the Ion Torrent PGM reads (miRNA sequences) of adult and neonate *C. s. simus* have been archived as Standard Flowgram Format (sff) files with the NCBI Sequence Read Archive (SRA) (http://www.ncbi.nlm.nih.gov/sra?term=SRA051956) under accession codes SRX143982 and SRX143985, SRX143983 and SRX143984, respectively.

### Animal ethics

Procedures for snake maintenance, sacrifice, and gland extraction in this study followed the Quality Management Protocols from the Instituto Clodomiro Picado, University of Costa Rica, and comply the Animal Welfare Law #7451, chapters II and III, and the Biodiversity Law, Decree 7788, Republic of Costa Rica. Research permission was allowed under Resolution ACG- SINAC- PI-012-2010 (Ministry of Environment of Costa Rica).

## Competing interests

The authors declare that they have no competing interests

## Authors’ contributions

AG, FB, SR and DC were responsible for all stages of animal care and venom extraction. FB, MS and AG dissected the venom glands. AP and LS prepared the RNA samples for 454 and Ion Torrent sequencing. All co-authors (JD, AP, LS, AG, FB, SR, DC, MS, YA, JMG, JJC) analyzed the data and participated in data interpretation and discussion of the results, as well as in revising the article drafted by JJC. All co-authors have seen, reviewed, and approved the final version of the manuscript.

## Supplementary Material

Additional file 1: Table S1 RepeatMasker usage results and features of the sequence elements masked in the adult *C. s. simus* venom gland transcriptomes analyzed. **Table S2.** RepeatMasker usage results and features of the sequence elements masked in the adult *C. s. simus* venom gland transcriptomes analyzed. Click here for file

Additional file 2: Table S3 Relative abundances of the different venom protein family hits in the venom gland transcriptomes of newborn and adult *C. s. simus*. **Table S4.** NoiSeq computed expression profile of highly expressed (>100 counts) miRNAs in the venom gland transcriptome listed by decreasing newborn (N) to adult (A) expression ratio. **Table S5.** NoiSeq computed expression profile of highly expressed (>100 counts) miRNAs in the venom gland transcriptome listed by decreasing adult (A) to newborn (N) expression ratio. **Table S6.** MiRanda predicted miRNAs complementary of 3'-UTR loci of 454 *C. s. simus* venom transcripts. Click here for file

Additional file 3: Figure S1 Multiple alignment of transcript-deduced amino acid sequences of PLA_2_ molecules. **Figure S2.** Multiple alignment of transcript-deduced serine proteinase amino acid sequences. **Figure S3.** Multiple alignment of transcript-deduced amino acid sequences of snake venom metalloproteinases. **Figure S4.** Predicted targets for the miRNAs displayed in Figure 7, showing their Watson-Crick pairing to target 3'-UTR loci of PLA_2_ and SVMP 454 transcripts, and the corresponding binding energy calculated by MapMi. Click here for file
