# Supplementary material for: Integrated “omics” profiling indicates that miRNAs are modulators of the ontogenetic venom composition shift in the Central American rattlesnake, Crotalus simus simus
Source: BMC Genomics. 2013 Apr 10;14:234. doi: 10.1186/1471-2164-14-234 (PMC3660174; doi:10.1186/1471-2164-14-234)
Supplement: Additional file 1: Table S1 — RepeatMasker usage results and features of the sequence elements masked in the adult C. s. simus venom gland transcriptomes analyzed. Table S2. RepeatMasker usage results and features of the sequence elements masked in the adult C. s. simus venom gland transcriptomes analyzed. [file 1471-2164-14-234-S1.pdf]

Table S1. RepeatMasker usage results and features of the sequence elements masked in the adult *C. s. simus* venom gland transcriptomes.Total contigs analyzed: 6484; Total length of sequences: 5043749 nucleotides; GC level: 41.81% number of bases masked: 97204 (1.93% of the total adult *C. s. simus* venom gland transcriptome).

	**number of elements**	**nucleotides**	**% contig sequences**
**Retroelements**	297	44474	0.88
SINEs:	62	7243	0.14
Penelope	24	2135	0.04
LINEs:	159	29857	0.59
CRE/SLACS	0	0	0.00
L2/CR1/Rex	95	15861	0.31
R1/LOA/Jockey	0	0	0.00
R2/R4/NeSL	6	1580	0.03
RTE/Bov-B	26	8435	0.17
L1/CIN4	8	1846	0.04
LTR elements:	76	7374	0.15
BEL/Pao	1	84	0.00
Ty1/Copia	0	0	0.00
Gypsy/DIRS1	17	1347	0.03
Retroviral	56	5859	0.12
**DNA transposons**	148	18708	0.37
hobo-Activator	94	11341	0.22
Tc1-IS630-Pogo	40	6558	0.13
En-Spm	0	0	0.00
MuDR-IS905	0	0	0.00
PiggyBac	2	103	0.00
Tourist/Harbinger	1	136	0.00
Other (Mirage, P-element, Transib)	0	0	0.00
**Rolling-circles**	0	0	0.00
**Unclassified**:	3	316	0.01
**Total interspersed repeats**:		63498	1.26
**Small RNA**:	18	5082	0.10
**Satellites**:	7	1177	0.02
**Simple repeats**:	275	11819	0.23
**Low complexity**:	432	15728	0.31

Table S2. RepeatMasker usage results and features of the sequence elements masked in the neonate *C. s. simus* venom gland transcriptomes.Total contigs analyzed: 6047; Total length of sequences: 3391553 nucleotides; GC level: 42.1%; number of bases masked: 65742 (1.94% of the total newborn *C. s. simus* venom gland transcriptome).

	**number of elements**	**nucleotides**	**% contig sequences**
**Retroelements**	189	28724	0.85
SINEs:	33	3873	0.11
Penelope	12	929	0.03
LINEs:	95	18524	0.55
CRE/SLACS	0	0	0.00
L2/CR1/Rex	59	9771	0.29
R1/LOA/Jockey	0	0	0.00
R2/R4/NeSL	3	834	0.02
RTE/Bov-B	16	6197	0.18
L1/CIN4	6	846	0.02
LTR elements:	61	6327	0.19
BEL/Pao	3	377	0.01
Ty1/Copia	0	0	0.00
Gypsy/DIRS1	17	2063	0.06
Retroviral	39	3803	0.11
**DNA transposons**	96	9494	0.28
hobo-Activator	63	5348	0.16
Tc1-IS630-Pogo	22	3686	0.11
En-Spm	0	0	0.00
MuDR-IS905	0	0	0.00
PiggyBac	1	55	0.00
Tourist/Harbinger	0	0	0.00
Other (Mirage, P-element, Transib)	0	0	0.00
**Rolling-circles**	0	0	0.00
**Unclassified**:	2	157	0.00
**Total interspersed repeats**:		38375	1.13
**Small RNA**:	50	11190	0.33
**Satellites**:	6	965	0.03
**Simple repeats**:	172	6799	0.20
**Low complexity**:	221	8557	0.25
