# Supplementary material for: Integrated “omics” profiling indicates that miRNAs are modulators of the ontogenetic venom composition shift in the Central American rattlesnake, Crotalus simus simus
Source: BMC Genomics. 2013 Apr 10;14:234. doi: 10.1186/1471-2164-14-234 (PMC3660174; doi:10.1186/1471-2164-14-234)
Supplement: Additional file 2: Table S3 — Relative abundances of the different venom protein family hits in the venom gland transcriptomes of newborn and adult C. s. simus. Table S4. NoiSeq computed expression profile of highly expressed (>100 counts) miRNAs in the venom gland transcriptome listed by decreasing newborn (N) to adult (A) expression ratio. Table S5. NoiSeq computed expression profile of highly expressed (>100 counts) miRNAs in the venom gland transcriptome listed by decreasing adult (A) to newborn (N) expression ratio. Table S6. MiRanda predicted miRNAs complementary of 3'-UTR loci of 454 C. s. simus venom transcripts. [file 1471-2164-14-234-S2.pdf]

Table S3

Relative abundances of the different venom protein family hits in the venom gland transcriptomes of newborn and adult *C. s. simus*. Abbreviations: 5'-NTase, 5'-nucleotidase; BPP, bradykinin potentiating peptide; CRISP, cysteine-rich secretory protein; CTL, C-type lectin-like protein; GC, glutaminyl cyclase; HYA, hyaluronidase; KUN, Kunitz-type inhibitor; LAO, L-amino acid oxidase; NGF, nerve growth factor; OHA, ohanin; PDE, phosphodiesterase; PLA_2, phospholipase A_2; SVMP, snake venom metalloproteinase; CRO, crotamine; SP, serine proteinase; 3FTX, three-finger toxin; VEGF, vascular endothelial growth factor.

	Newborn				Adult			
	Contigs	Reads	Singletons	%	Contigs	Reads	Singletons	%
5'-NTase	2	411	1	0.04	3	465	3	0.09
BPP	6	1,900	5	0.18	9	675	10	0.13
CRISP	5	76	0	0.01	0	0	1	0
CTL	8	591	7	0.06	18	1,969	6	0.38
GC	1	143	2	0.01	3	348	3	0.07
HYA	3	416	8	0.04	5	395	2	0.08

KUN	4	61	0	0.01	4	344	4	0.07
LAO	1	1,300	2	0.12	3	1,582	10	0.31
NGF	3	1,072	0	0.10	7	3,192	8	0.62
OHA	3	110	26	0.01	5	81	68	0.03
PDE	1	308	0	0.03	1	77	0	0.01
PLA_2	81	161,494	72	15.29	36	29,721	29	5.75
SVMP	92	129,838	100	12.30	123	161,789	87	31.27
CRO	0	0	1	0	1	7	2	0.001
SP	438	756,185	278	71.58	201	316,882	136	61.24
3FTX	4	44	18	0.01	5	93	49	0.03
VEGF	6	2,792	6	0.26	7	38	13	0.01

Table S4

NoiSeq computed expression profile of highly expressed miRNAs in the venom gland transcriptome listed by decreasing newborn (N) to adult (A) expression ratio.

**Cluster Newborn**	**Cluster Adult**	**Counts Newborn**	**Counts Adult**	**Ratio N / A**	**Sequence**
Cluster 120	Cluster 713	1,002	123	8.14	GTGGGTTCCGGGAGGGGACCGCCG
Cluster 804	Cluster 32637	456	109	4.18	TGAGGTAGTAGGTTGTATAGTTA
Cluster 542	Cluster 3288	3,582	901	3.97	ACGCGGGAGACCGGGGTTCGA
Cluster 462	Cluster 2747	819	221	3.70	TGGGTTCGCCCCGAGAGAGGGGCCCGCGC
Cluster 752	Cluster 620	415	115	3.60	AGCTGAAGAGGAAAACAACACTTTCCTTAGTGACACC
Cluster 2675	Cluster 2609	582	165	3.52	TGGAGCCGGGCGTGGAATGCGAGACGCCTAG
Cluster 825	Cluster 434	430	149	2.88	GGACCCGAAAGATGGTGAACTATGCCTGGGCA
Cluster 253	Cluster 134	4,717	1,747	2.7	AGCTGAAGAGGAAAACAACACTTTCCTTAGTGACCA
Cluster 1867	Cluster 1923	315	125	2.52	AGAGTGTTCAAAGCAGG
Cluster 5022	Cluster 4379	251	100	2.51	CCCGCGGAGCAGAAGGGCA
Cluster 13195	Cluster 19265	380	155	2.45	AGTGACGCGCATGAATGG

Cluster 499	Cluster 12083	237	100	2.37	TCACGCGGGAGACCGGGGTTCGA
Cluster 1	Cluster 86	995	424	2.34	CTGATCGTTTTTTCACTTACCCGGTGAGGCGGGGGGGCA
Cluster 11329	Cluster 37134	1,728	748	2.31	ATCCCACTTCTGACA
Cluster 84	Cluster 125	284	124	2.29	GTGCGCCTTGAAGCCTAG
Cluster 1825	Cluster 20	260	119	2.18	CGAAAGATGGTGAACTATGCCTGGGC
Cluster 763	Cluster 1537	314	150	2.09	GGCTGGTCCGATTGCAGTGGT
Cluster 875	Cluster 13	427	211	2.02	GCGGCGATTCCGGAGGCGAG
Cluster 9	Cluster 17	408	207	1.97	GCTGCAGACGAAAGCC
Cluster 18066	Cluster 23837	194	101	1.92	GCGTAGCCTGAGTAAGACAGCG
Cluster 14228	Cluster 11523	337	180	1.87	TGAGGTAGTAGATTGTATAGTTA
Cluster 5857	Cluster 2817	301	163	1.84	GGATCCCGAGGCCTCCGAGCGGAGGGCGCAC
Cluster 1358	Cluster 17802	271	148	1.83	CACGCGGGAGACCGGGGTTCGA
Cluster 1030	Cluster 980	176	100	1.76	CTAGCGAAACCACAGCCA
Cluster 3503	Cluster 3878	2,358	1,383	1.70	GCATTGGTGGTTCAGTGGTAGAATTCTCGCC
Cluster 1366	Cluster 789	641	378	1.69	AACGAGAACTTTGAAGGCCGA
Cluster 322	Cluster 1316	377	229	1.64	ACGAGAACTTTGAAGGCCGA
Cluster 4002	Cluster 5732	173	116	1.49	GACACGGAAAGGATTGACAGA
Cluster 15933	Cluster 6108	297	201	1.47	CGCGGGAAATGTGGCGTACGGAAGA
Cluster 1162	Cluster 1993	670	459	1.45	CGCGGGAAATGTGGCGTA
Cluster 2302	Cluster 22081	239	164	1.45	CTGATCGTTTTTTCACTTACCCGGA

Cluster 19406	Cluster 36092	148	102	1.45	GCAGTCCATGGGCATATACA
Cluster 769	Cluster 664	448	309	1.44	CCGACCATAAACGATGCCGACTAGCGA
Cluster 276	Cluster 364	146	102	1.43	TGACGTGCAAATCGGTCGTCCGACCTGGGTA
Cluster 12083	Cluster 14408	144	108	1.33	CGTGCATTTATCAGACCAAAACCAACCA
Cluster 1943	Cluster 7144	156	124	1.25	GGACCGGCGCAAGACGAC
Cluster 15116	Cluster 18871	166	137	1.21	GTAGAATAAGTGGGAGG
Cluster 411	Cluster 168	260	216	1.20	TCCGACCATAAACGATGCCGACTAGCGA
Cluster 1528	Cluster 810	402	335	1.20	TAAGGCGCCCGATGCCGACGCTCATCAGAC

Table S5

NoiSeq computed expression profile of highly expressed miRNAs in the venom gland transcriptome listed by decreasing adult (A) to newborn (N) expression ratio.

**Cluster Newborn**	**Cluster Adult**	**Counts Newborn**	**Counts Adult**	**Ratio A / N**	**Sequence**
Cluster 4562	Cluster 3477	255	3,420	13.41	TAGCTTATCAGACTGATGTTG
Cluster 24153	Cluster 39368	411	4,067	9.89	TCTCCCAACCTTGTACCAGTA
Cluster 2064	Cluster 1899	104	937	9	CGCGGTGGGACGCGGGAAATGTGGCGTACGGA
Cluster 5420	Cluster 9100	464	3,621	7.80	GAGTAAGACAGCGAGACCCAGA
Cluster 4648	Cluster 4753	133	965	7.25	AGGCGTCCAGTGCGGTAACGCGA
Cluster 528	Cluster 234	718	5,110	7.11	ATCACATTGCCAGGGATTACCA
Cluster 20610	Cluster 30365	524	3,401	6.49	CAACGGAATCCCAAAAGCAGCTA
Cluster 20407	Cluster 27405	395	2,414	6.11	AACCCGTAGATCCGAACTTGCGA
Cluster 2643	Cluster 3504	178	1,077	6.05	TTCAAGTAATCCAGGATAGGCA
Cluster 9004	Cluster 11537	420	2,409	5.73	CGTCCAGTGCGGTAACGCGAC
Cluster 3877	Cluster 3156	142	786	5.53	TCCCATATGGTCTAGCGGTTAGGATTCCTGGTA

Cluster 467	Cluster 308	341	1,771	5.19	GCTGCCGGTGAAATACCACTA
Cluster 3989	Cluster 4834	147	740	5.03	CATCTAAGGCTAAATACCGGCACGA
Cluster 4317	Cluster 6564	1,504	7,252	4.82	GCGCCGCTGGTGTAGTGGTATCATGCA
Cluster 14	Cluster 104	212	984	4.64	TGGCCCTTGAAAATCCGGGGGAGATGG
Cluster 113	Cluster 404	256	1,180	4.60	GCCGACAGGTGCGGGTA
Cluster 422	Cluster 2215	1,532	6,707	4.37	TCTCCCAACCTTGTACCAGTG
Cluster 17466	Cluster 24163	1,307	5,623	4.30	TGGCTCAGTTCAGCAGGAACAGTA
Cluster 18109	Cluster 32713	125	528	4.22	CAGTGCAATGTAAAAAGGGCAT
Cluster 1979	Cluster 983	114	479	4.20	CCGCGGTGGGACGCGGGAAATGTGGCGTACGG
Cluster 5185	Cluster 8848	602	2,504	4.15	GTCCAGTGCGGTAACGCGACCGA
Cluster 22352	Cluster 33768	301	1,200	3.98	CAACGGAATCCCAAAAGCAGCA
Cluster 22230	Cluster 35698	141	533	3.78	TCTCCCAACCCTTGTACCAGTA
Cluster 176	Cluster 1597	163	604	3.70	CGACAGGTGCGGGTA
Cluster 3894	Cluster 69	122	438	3.59	GGTTCCATAGTGTAGTGGTTATCACGTCTGC
Cluster 1267	Cluster 8551	271	990	3.54	CAACGGAATCCCAAAAGCAGCTG
Cluster 4274	Cluster 1269	134	450	3.35	GCGAGGCGTCCAGTGCGGTAACGCGAC
Cluster 8665	Cluster 20781	753	2509	3.33	TTCAAGTAATCCAGGATAGGCTA
Cluster 3103	Cluster 26193	1,005	3,223	3.20	TAGCAGCACGTAAATACTGGAG

Table S6

MiRanda predicted miRNAs complementary of 3'-UTR loci of 454 *C. s. simus* venom transcripts

Newborn Cluster ID	Contig Map	Best Blast Hit Description	Adult Cluster ID	Contig Map	Best Blast Hit Description
New 752	contig00479	M89784 | Atrolysin e	Ad 620	contig00703	AF378289 | SVMP Clone Mol(c)
	contig00484	M89784 | Atrolysin e		contig00819	M89784 | Atrolysin e
	contig03129	AB059571 | Elegantin-1a		contig00790	D28871| Prepro-halystatin 2 & 3
	contig03089	GQ451440 | SVMP VMP-II		contig00791	HM443640 | SVMP MP_IIx2
	contig00483	M89784 | Atrolysin e		contig00808	GQ451439 | SVMP VMP-III
	contig03079	GQ451440 | SVMP VMP-II		contig00691	GQ451439 | SVMP VMP-III
New 253	contig00479	M89784 | Atrolysin e	Ad 134	contig00703	AF378289 | SVMP Clone Mol(c)
	contig00484	M89784 | Atrolysin e		contig00819	M89784 | Atrolysin e
	contig03129	AB059571 | Elegantin-1a		contig00790	D28871 | Prepro-halystatin 2 & 3
	contig00483	M89784| Atrolysin e		contig00791	HM443640 | SVMP MP_IIx2
				contig00808	GQ451439 | SVMP VMP-III
				contig00691	GQ451439 | SVMP VMP-III

New 3503	contig03089	GQ451440 | SVMP VMP-II	Ad 2817	contig00886	M89784 | Atrolysin e
	contig03090	D28871 | Prepro-halystatin 2 & 3		contig00712	M89784 | Atrolysin e
	contig03078	HM443642 | SVMP MP_IIa		contig00964	GQ451443 | disint precursor
New 87	contig04061	GQ451441 | SVMP VMP-III	Ad 222	contig00886	M89784 | Atrolysin e
	contig03230	GQ451441 | SVMP VMP-III		contig00712	M89784 | Atrolysin e
	contig00479	M89784 | Atrolysin e		contig00703	AF378289 | SVMP Clone Mol(c)
	contig00484	M89784 | Atrolysin e		contig00819	M89784 | Atrolysin e
	contig03434	AB042840 | Vap-1		contig00936	AF117637 | SVMP MD2
				contig00699	U86634 | SVMP-disint-like
				contig00948	AF378290 | SVMP Clone Mol(d)
				contig00780	GQ451439 | SVMP VMP-III
New 166	contig00479	M89784 | Atrolysin e	Ad 44	contig00886	M89784 | Atrolysin e
	contig00484	M89784 | Atrolysin e		contig00712	M89784 | Atrolysin e
	contig03434	AB042840 | Vap-1		contig00703	AF378289 | SVMP Clone Mol(c)
				contig00819	M89784 | Atrolysin e
				contig00699	U86634 | SVMP-disint-like
				contig00948	AF378290 | SVMP Clone Mol(d)

	contig00780	GQ451439 | SVMP VMP-III
New 2681	contig03079	GQ451440 | SVMP VMP-II
	contig00775	U01237 | Atrolysin d
New 3708	contig03089	GQ451440 | SVMP VMP-II
	contig03090	D28871 | Prepro-halystatin 2 & 3
	contig03078	HM443642 | SVMP MP_IIa
New 20	contig00479	M89784 | Atrolysin e
	contig00484	M89784 | Atrolysin e
	contig03434	AB042840 | Vap 1
	contig01298	M89784 | Atrolysin e
	contig03228	GQ451441 | SVMP VMP-III
Ad 368/New 299	contig02859	X12603 | Crotalus durissus terrificus RNA for crotoxin B
	contig02858	X12603 | Crotalus durissus terrificus RNA for crotoxin B
Ad 1078/New 1849	contig01024	U01027 | Crotalus scutulatus scutulatus Mojave Toxin subunit gene
	contig00975	U01026 | Crotalus scutulatus scutulatus Mojave Toxin subunit gene
	contig00986	AF269131 | Crotalus atrox acidic phospholipase A2 precursor mRNA
	contig00974	U01026 | Crotalus scutulatus scutulatus Mojave Toxin subunit gene
Ad 2166	contig02859	X12603 | Crotalus durissus terrificus RNA for crotoxin B
	contig02858	X12603 | Crotalus durissus terrificus RNA for crotoxin B

	contig01024	U01027 | Crotalus scutulatus scutulatus Mojave Toxin subunit gene
	contig01004	U01027 | Crotalus scutulatus scutulatus Mojave Toxin subunit gene
New 4393/Ad 3416	contig03089	GQ451440 | Crotalus viridis viridis metalloproteinase VMP-II precursor
	contig03090	D28871 | AGKAHB Gloydius halys gene for prepro-halystatin 2 and 3
	contig03078	HM443642 | Bothrops neuwiedi MP_IIa SVMP precursor
New 2578	contig03130	U01234 | CRLPREHTA Crotalus atrox hemorrhagic toxin a, atrolysin a (Ht-a)
	contig03228	GQ451441 | Agkistrodon piscivorus leucostoma metalloproteinase VMP-III
	contig03079	GQ451440 | Crotalus viridis viridis metalloproteinase VMP-II precursor
	contig03128	GQ451443 | Agkistrodon piscivorus leucostoma disintegrin precursor
