# Supplementary material for: Integrated “omics” profiling indicates that miRNAs are modulators of the ontogenetic venom composition shift in the Central American rattlesnake, Crotalus simus simus
Source: BMC Genomics. 2013 Apr 10;14:234. doi: 10.1186/1471-2164-14-234 (PMC3660174; doi:10.1186/1471-2164-14-234)
Supplement: Additional file 3: Figure S1 — Multiple alignment of transcript-deduced amino acid sequences of PLA2 molecules. Figure S2. Multiple alignment of transcript-deduced serine proteinase amino acid sequences. Figure S3. Multiple alignment of transcript-deduced amino acid sequences of snake venom metalloproteinases. Figure S4. Predicted targets for the miRNAs displayed in Figure 7, showing their Watson-Crick pairing to target 3'-UTR loci of PLA2 and SVMP 454 transcripts, and the corresponding binding energy calculated by MapMi. [file 1471-2164-14-234-S3.pdf]

**Figure S1. Multiple alignment of transcript-deduced amino acid sequences of $PLA_2$ molecules.**

```
                                                                      **  *****
Cluster_0  MRALWIVAVLLVGVEGHLLQFNKMIKFETRKNAIPFYAFYGCYCGWGGRGRPKDATDRCCFVHDCCYGKLAKCNTKWDIY
Cluster_1  MRALWIVAVLLVGVEGSLVEFETLMMKIAGRSGISYYSSYGCYCGAGG--------DRCCFVHDCCYAKLTGCDPTTDVY
Cluster_2  MRALWIVAVLLVGVEGSLVEFETLMMKIAGRSGISYYSSYGCYCGAGGQGWPQDASDRCCFEHDCCYAKLTGCDPTTDVY
Cluster_3  ----------------------------------------------------------CCFEHDCCYAKLTGCDPTTDVY
Cluster_4  ----------------------------------------------------------CCFVHDCCYGKATDCNPKTVSY
Cluster_5  MRTLWIVAVLLVGVKGSLVEFGKMIKEETGKNPFPSYTFYGCYCGLGGQGRPKDATDRCCFVHDCCY-------------
Cluster_6  ----------------------------------------------------------CCFEHDCCYAKLTGCDPTTDVY
Cluster_7  MRALWIVAVLLV---------------GGER---------------------------CCRVHDCCYQALS---------
           1.......10........20........30........40........50........60........70........80


Cluster_0  PYSLKSGYITCGKGTWCEEQICECDRVAAECLRRSLSTYKYGYMFYPDSRCRGPSETC-
Cluster_1  TYRQEDGEIVCGGDDPCGTQICECDKAAAICFRISMDTYDYKYLQFSPENCQGESQPC-
Cluster_2  TYRQEDGGNRLWRGT--------------------------------------------
Cluster_3  TYRQEDGEIVCGGKGTWCEEQICECDRVAAECLRRSLSTYKYGYMFYPDSRCRGPSETC
Cluster_4  TYSEENGEIICGGDDPCGTQICECDKAAAICFRDNIPSYNNKYWLFPPKNCREEPEPC-
Cluster_5  -----------------------------------------------------------
Cluster_6  TY-----------KGTWCEEQICECDRVAAECLRRSLSTYKYGYMFYPDSRCRG-----
Cluster_7  ---------------------------------RRHCKPKMEKYFYSVRKDTVTC----
           ........90.......100.......110.......120.......130.........
```

**Figure S2. Multiple alignment of transcript-deduced serine proteinase amino acid sequences.**

```
Cluster_0  MVLIRVLANLLILQLSYAQKSSELIFGGRPCNRNEHRFLALVY---SDGNQCSGTLINKEWVLTAAHCEGKKK-XKIHLG   76
Cluster_1  MVLIRVLATLLILQLSYGQKSSELVIGGDECNINEHRSLALIY--ITSDGFCGGTLINKEWVLTAVHCDKGNM--LIFLG   76
Cluster_2  -----------------AQKSSELVIGGDECNINEHRSLALVY--ITSGFLCGGTLINKEWVLTAAHCDRGDI---LVLL   58
Cluster_3  -------------------------------NXNEHNFLVALYEYWSQSFLCGGTLINGEWVLTAAHCDRTHF--LIYVG   47
Cluster_4  MVLIRVLANLLILQLSYAQKSSELVIGGDECNINEHRFLVALYHYRSHTFLCGGILINKEWVLTAKHCDRKFM--YIRLG   78
Cluster_5  ------------------------------------------------------------------------KNFQMQLG    8
Cluster_6  MVLIRVLANLLILQLSY-QKSSELVIGGDECNINEHRFLVALY--DPDRFLCSGILLNEEWALTAAHCDRRNI--RIKLG   75
Cluster_7  MVLISVLANLLILQLSYAQKSAELIIGSDECNINEHRFLALMY---SDRFQCSGTLINEEWVLTAAHCDTLLD-------   70
Cluster_8  --------------------------------------------------------------------------------
Cluster_9  MVLIRVLANLLILQLSYAQKSSELVVGGDECNINEHRSLVAIF--NSTEFFCSGTLINQEWVVTAAHCDSTNF--KMKLG   76
Cluster_10 ------------------------------------------------------------------------KKLKLQFG    8
Cluster_11 --------------------------------------------------------------------------------
Cluster_12 ------------------------------------------------GFLCAGTLINQEWVLTAAHCKIKNF--QIKLG   30
Cluster_13 --------------------------------------------------------------------------------
Cluster_14 MVLIRVLANLLILQLSYAQKSSELVIGGDECNINEHNFLVALYEYWSQSFLCGGTLINGEWVLTAAHCDRTHF--LIYVG   78
Cluster_15 --------------------------------------------------------------------------------
Cluster_16 -----------------AQKSSELIFGGRPCNRNEHRFLALVY---SDGNQCSGTLINKEWVLTAAHCE-----------   49
Cluster_17 MGLIRVLANLLILQLSYGKSSSELVIGGDECNINEHRSLALVYITSGFXCG-----------------------------   51
Cluster_18 ------------------QKSSELVVGGHPCNINEHRSLVVLF--NSSRFLCAGTLINEQWVLTAAHCDSKNF-------   53
Cluster_19 ------------------QKSSELIIGGEECNINEHRFLVALYTFRSKRFHCSGTLINQEWVLTAAHCD-----------   51
           1.......10........20........30........40........50........60........70........80

Cluster_0  VHSKKVPNKDKQTRVAKEKFFCVSSKNYTKWNKDIMLIRLDRSVSNSEHIAPLKLPSSSPSVGSVCRIMGWGTISPTEVI  156
Cluster_1  MHSLEVPNKDVQKRVAKEKFICPNRKKDDKKDKDIVLIRLDSPVNNSTHIAPLSLPSSPPSVGSVCRIMGWGAITSPNVT  156
Cluster_2  GVHRLKDVQTGVSKDVQTRVAKEKFICPNRKKKDIMLIRMDSPVNISTHIAPLSLPSNPPSVGSVCRIMGWGAITSPNVT  138
Cluster_3  VHDRSVQFDKEQRRFPKEKYVF----NFTEWDKDIMLIRLNKPVRNNEHIAPLSLPSSPPSVGSVCRVMGWGQTTSPQNT  123
Cluster_4  MHNKNVKFDDDQRRSSKKKYFFRCHNNFTRWDKDIMLIRLNQPVSYSEHIAPLSLPSSPPSVGSVCRVMGWGQTTSPQRD  158
Cluster_5  VHSKKVLNEDEQTRDPKEKFICPNKKKDDEKDKDIMLIRMDSPVSNSEHIAPLSLPSSPPSVGSVCRIMGWGTISPTKET   88
Cluster_6  MHSKTVPNEDEQTRAPKEKFFCLSSKNYTLWDKDIMLIRLDSPVSNSEHIAPFSLPSSPPSVGSVCRIMGWG-----KET  150
Cluster_7  --------------------------------------------------------------------------------   70
Cluster_8  -------------RVPKEKFFCLSSKTYTKWDKDIMLIRLKRPVNNSAHIATLSLPSNPPSLGSVCRIMGWGTIT-----   62
Cluster_9  VHSKKVPNEDEQTRNPKEKFFCPNKKKDDVLDKDIMLIKLDSPVSNSEHIMPLSLPSSPPSVGSVCHIMGWGSITPI---  153
Cluster_10 LHSKNVPNKDKQTRVPKEKFFCLSSKNNKEWDKDIMLIRLNRPVNNSKHIAPLSLPSKPPSQDTVCNIMGWGTISPTKET   88
Cluster_11 -------------------------------DKDIMLIKLDSPVSNSEHIMPLSLPSSPPSVGSVCHIMGWGSITPIEXT   49
Cluster_12 VHSKKVRNEDEQTRDPKEKFFCLGSKTNNEWEKDIMLIRLNNPVSNSAHIAPLSLPSSPPSVGXLCRIMGWGTISSTKEI  110
Cluster_13 -------------------------------------------------------------VGSVCHIMGWGSITPIEKA   19
Cluster_14 VHDRSVQFDKEQRRFPKEKLLFNCGNN-----------------------------------------------------  105
Cluster_15 --------------------------------------------------------------------------------
Cluster_16 ----------KQTRVAKEKFFCVSSKNYTKWNKDI---------------------------------------------   74
Cluster_17 --------------------------------------------------------------------------------   51
Cluster_18 --------------------------------------------------------------------------------   53
Cluster_19 --------------------------------------------------------------------------------   51
           ........90.......100.......110.......120.......130.......140.......150.......160

Cluster_0  LPDVPHCANINLLNYSECRAAYPEYGLPATSRTLCAGILEGGKDTCVGDSGGPLICNGQFQGIASWGST-LCGYVREPAL  235
Cluster_1  LPAVPHCANINIVRNALCRKAYAG--LPATSRTLCAGILQGGKDACVGDSGGPLICNGQIQGIVSGGGGHPCGQSLKPGF  234
Cluster_2  LPGVPHCADINIFDYEVCRAAKPE--LPVTSRTLCAGILEGGKGSC-GDSGGPLICNGEIQGIVSW----GCAQPREPGH  211
Cluster_3  LPNVPHCANINLLDYEVCRTALPQLRLPATSRILCAGVLEGGIDTCNRDSGGPLICNGEFQGIVFWG---PCAQPDKPAL  200
Cluster_4  SGGPLICNGEXQGILSVGGDPCAQ--SHVPALYTKVFDYNDWIQSIIAGNTAATCP------------------------  212
Cluster_5  YPDVPHCANINILDHAVCRAAYPW--DPVTSTTLCAGTQQGGKDTCGADSGGPLICNGQFQGIVSWGGH-PCGQAREPGV  165
Cluster_6  YPDVPHCANINLLDYEVCLAAY----------------------------------------------------------  172
Cluster_7  ---VPHCADINILDYEVCQAAYPE--LPATRRTLCAGILEGGKDS---DSGGPLICNGQFQGIVSWGAD-TCAQPREPGL  141
Cluster_8  YPDVPHCANINILDYEVCRAAHGX--LPATSRTLCAGILEGGKDTCRGDSGGPLICNEVIQGIVSWG---------KPGV  131
Cluster_9  --------------------------------------------------------------------------------  153
Cluster_10 YPDVPHCANINILDHAVCRAIYPG--LLEKSRVLCAGVLEGGKDTCGGDS------------------------------  136
Cluster_11 LPDVPYCANIKLLDDAVCKPPYPE--LPATSRTLCAGILEGGKDTCGGDSGGPLICNGQFQGIVFYGAH-PCGQAPKPGV  126
Cluster_12 YPNVPRCA------------------------------------------------------------------------  118
Cluster_13 LPDVPYCANIKLLDDAV---PYPE--LPATSRTLCAGILEGGKDTCGGDSGGPLICNGQFQGIVFYGAH-PCGQAPKPGV   93
Cluster_14 --------------------------------------------------------------------------------  105
Cluster_15 ----------------VCRAAYPW--WPVTTRILCAGILEGGKDSCQGDSGGPLICNGQFQGIVSWXAH-PCGRRLNPGF   61
Cluster_16 --------------------------------------------------------------------------------   74
Cluster_17 --------------------------------------------------------------------------------   51
Cluster_18 --------------------------------------------------------------------------------   53
Cluster_19 --------------------------------------------------------------------------------   51
           .......170.......180.......190.......200.......210.......220.......230.......240
```

```
Cluster_0  YTKVFDHLDWIQSIIAGNTDATCP   259
Cluster_1  YTKVFD------------------   240
Cluster_2  YTKVFDYTEWIQSIIAGNTDXTCP   235
Cluster_3  YSKVFDHLDWIQSIIAGNTIVNCP   224
Cluster_4  ------------------------   212
Cluster_5  YVKVFDHLDWIQSIIAGNTTVNCP   189
Cluster_6  ------------------------   172
Cluster_7  YTKVFDYTDWIKSIISGNTDVT--   163
Cluster_8  YTXVFDYTXWIQSIIAGNTDATXP   155
Cluster_9  ------------------------   153
Cluster_10 ------------------------   136
Cluster_11 YT----------------------   128
Cluster_12 ------------------------   118
Cluster_13 YTKVFDYNDWIQSIIAGNTAATCP   117
Cluster_14 ------------------------   105
Cluster_15 YTKVFDYIDWIQSIIAGNTTVTCP    85
Cluster_16 ------------------------    74
Cluster_17 ------------------------    51
Cluster_18 ------------------------    53
Cluster_19 ------------------------    51
           .......250.......260....
```

**Figure S3. Multiple alignment of transcript-deduced amino acid sequences of snake venom metalloproteinases.**

```
Cluster_0  MIQVLLVTICLAVFPYQGSSIILESGNVNDYEVVYPRKVTALPKGAVQPKYEDTMQYEFKVNGEPVVLHLEKN--KGLFS   78
Cluster_1  MXXVLLXTICLAAFPYQXXSXILESXNXNDYEVXYPRKVTXLPKGAVQPKYEDXMQYEXXVNGEPVVLXLEKNKTKGLFS   80
Cluster_2  MIQVLLVTICLAVFPYQGSSIILESGXVNDYEVVYPRKVTALPKGAVQPKYEDXMQYELKVNGEPVVLXLEKN--KXLFS   78
Cluster_3  --------------------------------------------------------------------------------
Cluster_4  --------------------------------------------------------------------------------
Cluster_5  --------------------------------------------------------------------------------
Cluster_6  --------------------------------------------------------------------------------
Cluster_7  -----------------------------------------------------------------------------LFS    3
Cluster_8  --------------------------------------------------------------------------------
Cluster_9  --------------------------------------------------------------------------------
Cluster_10 --------------------------------------------------------------------------------
Cluster_11 --------------------------------------------------------------------------------
Cluster_12 --------------------------------------------------------------------------------
Cluster_13 --------------------------------------------------------------------------------
Cluster_14 --------------------------------------------------------------------------------
Cluster_15 --------------------------------------------------------------------------------
Cluster_16 --------------------------------------------------------------------------------
Cluster_17 --------------------------------------------------------------------------------
Cluster_18 --------------------------------------------------------------------------------
Cluster_19 --------------------------------------------------------------------------------
Cluster_20 --------------------------------------------------------------------------------
           1.......10........20........30........40........50........60........70........80

Cluster_0  EDYSETHYSPDGREITTYPPVEDHCYYHGRIQNDADSTASISACNGLKGHFKLQGEMYLIEPLKLXDS---EAHAVYKYE  155
Cluster_1  EDYSETHYSPDGREITTYPTIEDHCYYHGRIENDADSTASISACNGLKGHFKLQGEIYLIEPLKLPDSAVHAAHAVYKYE  160
Cluster_2  KDYXETHYSPDGRKITTNPSVEDHCYYHGRIENDADSTASISACNGLKGHFKLQGEMYLIEPLELSDS---EAHAVFKLE  155
Cluster_3  -----------GRKITTNPSVEDHCYYRGRIENDADSTASISACNGLKGHFKLQGEMYLIEPLELSDS---EAHAVFKLE   66
Cluster_4  --------------------------------------------------------------------------------
Cluster_5  --------------------------------------------------------------------------------
Cluster_6  --------------------------------------------------------------------------------
Cluster_7  EDYSETHYSPDGREITTYPPVEDHCYYHGRIQNDADSTASISACNGLKGHFKLQGEMYLIEPLKLPDS---EAHAVFKYE   80
Cluster_8  --------------------------------------------------------------------------------
Cluster_9  --------------------------------------------------------------------------------
Cluster_10 --------------------------------------------------------------------------------
Cluster_11 --------------------------------------------------------------------------------
Cluster_12 --------------------------------------------------------------------------------
Cluster_13 --------------------------------------------------------------------------------
Cluster_14 --------------------------------------------------------------------------------
Cluster_15 --------------------------------------------------------------------------------
Cluster_16 --------------------------------------------------------------------------------
Cluster_17 --------------------------------------------------------------------------------
Cluster_18 --------------------------------------------------------------------------------
Cluster_19 --------------------------------------------------------------------------------
Cluster_20 --------------------------------------------------------------------------------
           ........90.......100.......110.......120.......130.......140.......150.......160

Cluster_0  NVEKEDEAPKMCGVTXNWESYEPIKKASQLNLTPEQXRYLSTKKYIELVIVADNVMVKKYTSNSTAIRTRIYACVNXLNL  235
Cluster_1  NVEKEDEAPKMCGVTQNWESYEPIKKTSQSTLTPEQQRYLNAKKYVKLFLVADYIMYLKYGRNLTAVRTRMYDIVNVITP  240
Cluster_2  NVEKEDEAPKMCGVTQNWESYEPIKKASXLXVTAEHQKY-NPFRFVELVLVVDKAMVTKNNDDLDKIKTRMYELANTVND  234
Cluster_3  NVEKEDEGPKMCGVTQNWESYEPIKKASDLNLNPDQQNL--PQRYIELVIVADHRVFMKYNSDLNIIRTRVHEIVNFING  144
Cluster_4  --------------------------------------------------------------------------------
Cluster_5  -----------------WESNEPIKKASHLNLNPEHQRY------VEIVIVVDHGMFTKYNGDSDKIRQRVHQMVNIMKE   57
Cluster_6  --------------------------------------------------------------------------------
Cluster_7  NVEKEDEAPKMCGVTQNWESYEPIKKASHLNLNPEQQRYLNNFRYLELVIVVDYKMYTKYNGNLIIIRAWVYEIFNTINE  160
Cluster_8  --------------------------------------------------------------------------------
Cluster_9  --------------------------------------------------------------------------------
Cluster_10 -------------VTQNWKSYEPIKKASQLNLTPEQQRF--PQRYIELIIVADHRMFTKYNSNLNTIRIWVHEIVNTINV   65
Cluster_11 --------------------------------------------------------------------------------
Cluster_12 --------------------------------------------------------------------------------
Cluster_13 --------------------------------------------------------------------------------
Cluster_14 --------------------------------------------------------------------------------
Cluster_15 --------------------------------------------------------------------------------
Cluster_16 --------------------------------------------------------------------------------
Cluster_17 --------------------------------------------------------------------------------
Cluster_18 --------------------------------------------------------------------------------
Cluster_19 --------------------------------------------------------------------------------
Cluster_20 --------------------------------------------------------------------------------
           .......170.......180.......190.......200.......210.......220.......230.......240
```

```
Cluster_0  IYRAFNIHIALVGLEIWSNKDLIXVQSAAADTLKEFGDWREADLLKRKKHDNAQLLTAIDLDGLTIGLAHVGSMCDPKRS  315
Cluster_1  IYHRMNIHVALVGLEIWSNTDKIIVQSSADVTLDLFAKWRATDLLSRKSHDNAQLLTGINFNGPTAGLGYLGGICNTMYS  320
Cluster_2  IYRYMYIHVALVGLEIWSNEDKITVKPEAGYTLNAFGEWRKTDLLTRKKHDNAQLLTAIDLDRVIGLAYVG---------  305
Cluster_3  FYRSLNIDVSLTDLEIWSDQDFITVQSSAENTLHSFGEWRKSVLLNRKRHDNAQLLTAIVLDKRTLGLAYLSSMCHPKHS  224
Cluster_4  ---------ALVGLEIWSHGDKIIVQSSADITLDLFGTWRATDLLRRKSHDNAQLLTSIDLDGPTIGLAYIGGICDPKXS   71
Cluster_5  SYSYMYIDIXLAGIEIWSNXDLINVQPAAPDTLNSFGEWRETDLLKRKSHDNAQLLTSIDFDGQTIGLAYIGGICDSKRS  137
Cluster_6  --------------------------------------------------------------------------------
Cluster_7  IFQRMNI-------------------------------------------------------------------------  167
Cluster_8  --------------------------------------------------------------------------------
Cluster_9  ------------------------------------------------------------------------SMCHPKRS    8
Cluster_10 FYRSLHIVVSLTDLEIWSNQDQINVQSAAADTLEAFGEWRETVLLNRISHDNAQLL------------------------  121
Cluster_11 ----------------------------------------------------------------TIGRAYMASMCEPKRS   16
Cluster_12 --------------------------------------------------------------------------------
Cluster_13 ----------------------------------------------------------------VIGKAYTGSMCDPRKS   16
Cluster_14 --------------------------------------------------------------------------------
Cluster_15 --------------------------------------------------------------------------------
Cluster_16 --------------------------------------------------------------------------------
Cluster_17 --------------------------------------------------------------------------------
Cluster_18 --------------------------------------------------------------------------------
Cluster_19 --------------------------------------------------------------------------------
Cluster_20 --------------------------------------------------------------------YEAEDSCFERNQ   12
           .......250.......260.......270.......280.......290.......300.......310.......320

Cluster_0  TGIVQDHNKVDVIVGITMAHELAHNLGMNHDGNQCNCGDKPCIMSEKLIFELGYRFSDCSRDEHWKYLIKKRPPCILNKP  395
Cluster_1  AGIVQDHSKIHHLVAIAVAHEMGHNLGMDHDKDTCTCGTRPCIMAGALSCEASFLFSDCSQKDHQEFLIKNMPQCILKKP  400
Cluster_2  --------------------------------------------------------------------------------  305
Cluster_3  VGIVQDHSPINLLMGVTMAHELGHNLGMKHDGDECLRGASLCIMRP----------------------------------  270
Cluster_4  TGVVQDFSPINFLVAXTMAHEMGHNLGMTHXEXYCSXGGXACIMSXVISPQPSKXFSXCSYIHYWXYINYXNPQCILNKP  151
Cluster_5  TGVXMDHS--NLRVAVTMTHELGHNLGIRHDTGSCSCGGYSCIMSPVISLQPSKYFSDCSYIQCWNFIMNQKPQCIPKKP  215
Cluster_6  --------------------------------------------------------------------------------
Cluster_7  --------------------------------------------------------------------------------  167
Cluster_8  --------------------------------------------------------------------------------
Cluster_9  TGIIQDYSPINLVVAVIMAHEMGHNLGIHHDSGYCSCGDYACIMRPEISPEPSTFFSNCSYFDCWDFIMNQNPECIVNEP   88
Cluster_10 --------------------------------------------------------------------------------  121
Cluster_11 TGIVQDHSPVNLLVAVTMAHEMGHNLGLHHDGKSCTCGDYICIMNC----------------------------------   62
Cluster_12 --------------------------------------------------------------------------------
Cluster_13 VGIVTDHSAINLWVAVTMAHELAHNLGISHDGNQCHCDANSCIMSEEL--------------------------------   64
Cluster_14 --------------------------------------------------------------------------------
Cluster_15 --------------------------------------------------------------------------------
Cluster_16 ------------------------------------------------SDEPSKYFSDCSYIQCWDFIMNQNPQCILKKP   32
Cluster_17 --------------------------------------------------------------------------------
Cluster_18 --------------------------------------------------------------------------------
Cluster_19 --------------------------------------------------------------------------------
Cluster_20 KGNYYGYCRK----------------------------------------------------------------------   22
           .......330.......340.......350.......360.......370.......380.......390.......400

Cluster_0  SITDIVSPPVCGNYFVELGEECDCGLPAHCQNPCCNATTCKLXPGTQCEDGXCCXXCXFXXXGTXCRXAXXECXXAESCT  475
Cluster_1  LKTDVVSPAVCGNYFVEVGEECDCGSPRTCRDPCCDAATCKLRQGAQCAEGLCCDQCRFKGAGTECRAAKDECDMADVCT  480
Cluster_2  --------------------------------------------------------------------------------  305
Cluster_3  --------------------------------------------------------------------------------  270
Cluster_4  LRTDIVSPPVSGNELLEAGEECDCGSP---RNPCCDAATCKLRPGAQCAEGLCCDHCRFTRAGKICRRARGDNPDDRCNG  228
Cluster_5  --------------------------------------------------------------------------------  215
Cluster_6  ----------------------------------CDAATCKLRQGAQCAEGLCCDQCRFKGAGTECRAAKDECDMADVCT   46
Cluster_7  --------------------------------------------------------------------------------  167
Cluster_8  ------------------------------------------------------------TAGTECRARRSECDIAENCT   20
Cluster_9  LGTDIISPPVCGNELLEVGEECDCGTPENCQNECCDAATCKLKSGSQCGHGDCCEQCK----------------------  146
Cluster_10 --------------------------------------------------------------------------------  121
Cluster_11 ------------------------------RDPCCDATTCKLHSWVECESGECCEQCR----------------------   90
Cluster_12 --------------------------------------TCKLKSGSQCGHGDCCEQCKFSKSGTECRASMSECDPAEHCT   42
Cluster_13 --------------------------------------------------------------------------------   64
Cluster_14 ----------------------------HCQNPCCNATTCKLKPGTQCEDGECCDRCQFKKGTVCRVARGGWNDDHCTGQ   52
Cluster_15 --------------------------------------------------------------------------------
Cluster_16 LRTDTVSTPVSGNEL-----------------------------------------------------------------   47
Cluster_17 --------------------------------------------------------RFMKKGTVCRVSLVNKNDDTCTGQ   24
Cluster_18 --------------------------------------------------------------------------------
Cluster_19 ----------------------------------CYATTCKLRPGSQCAEGMCCDQCR----------------------   24
Cluster_20 --------------------------------------------------------------------------------   22
           .......410.......420.......430.......440.......450.......460.......470.......480
```

```
Cluster_0  GQSADCPTDDFQRNGQPCLNNFGYCYNGKCPTLDDQCISFFGSSATVAPDVCFNLNLQGRGDFYCRRENTRIFPCAPQDK  555
Cluster_1  GRSTECTDRFQRNGQPCKNNNGYCYNGKCPIMADQCIALFGPGATVSQDACFQFNREGNHYGYCRKEQNTKIACEP----  556
Cluster_2  --------------------------------------------------------------------------------  305
Cluster_3  --------------------------------------------------------------------------------  270
Cluster_4  QSADCPRNRFHGDGMVCSNRQCVDVTTAY---------------------------------------------------  257
Cluster_5  --------------------------------------------------------------------------------  215
Cluster_6  GRSTECTDRFQRNGQPCKNNNGYCYNGKCPIMADQCIALFGPGATVSQDACFQFNREGNHYGYCRKEQNTKIACEPQDVK  126
Cluster_7  --------------------------------------------------------------------------------  167
Cluster_8  GQSDDCPTDDFHRNGQPCLNNFGYCYNGNCPILYHQCYAQFGPNATVGEDSCFEKNRIGNDYGYCTKENGRKIACAPQDV  100
Cluster_9  --------------------------------------------------------------------------------  146
Cluster_10 --------------------------------------------------------------------------------  121
Cluster_11 --------------------------------------------------------------------------------   90
Cluster_12 GQSSECPADVFHKNGQPCLDNYGYCYNGNCPIMYHQCYDLFGAD------------------------------------   86
Cluster_13 --------------------------------------------------------------------------------   64
Cluster_14 SGDCP---------------------------------------------------------------------------   57
Cluster_15 -------------------------------------------------------------------------------K    1
Cluster_16 --------------------------------------------------------------------------------   47
Cluster_17 SADCPGDGKVCSNGXCVDVITA----------------------------------------------------------   46
Cluster_18 -----------------------------------------------------------------------------DKK    3
Cluster_19 --------------------------------------------------------------------------------   24
Cluster_20 --------------------------------------------------------------------------------   22
           .......490.......500.......510.......520.......530.......540.......550.......560

Cluster_0  KCGRLFCVLGPTGNTISCQSIYSQSDLDTGMVALGTKCGDGRVCNSTRQCVDVNTVY  612
Cluster_1  ---------------------------------------------------------  556
Cluster_2  ---------------------------------------------------------  305
Cluster_3  ---------------------------------------------------------  270
Cluster_4  ---------------------------------------------------------  257
Cluster_5  ---------------------------------------------------------  215
Cluster_6  CGRLYCFPNSPENKNPCNIYYSPNDEDKGMVLPGSKCADGKACSNGQCVDVTTPY--  181
Cluster_7  ---------------------------------------------------------  167
Cluster_8  KCGRLYCSYHS--------NRNLCVSSSGIVEPGTKCGDGKVCSNGQCIDVTTAY--  147
Cluster_9  ---------------------------------------------------------  146
Cluster_10 ---------------------------------------------------------  121
Cluster_11 ---------------------------------------------------------   90
Cluster_12 ---------------------------------------------------------   86
Cluster_13 ---------------------------------------------------------   64
Cluster_14 ---------------------------------------------------------   57
Cluster_15 CGRLYCKDNSPGQNNPCKMFYSNEDEHKGMVLPGTKCADGKVCSNGHCVDVATAY--   56
Cluster_16 ---------------------------------------------------------   47
Cluster_17 ---------------------------------------------------------   46
Cluster_18 CGRLFCVQGPTGNKISCQSRAVPNDLNFGMVALGTKCGDGR----------------   44
Cluster_19 ---------------------------------------------------------   24
Cluster_20 ---------------------------------------------------------   22
           .......570.......580.......590.......600.......610.......
```

**Figure S4. Predicted targets for the miRNAs displayed in Table 6, showing their Watson-Crick pairing to target 3'-UTR loci of $PLA_2$ and SVMP 454 transcripts, and the corresponding binding energy calculated by MapMi.**

**Ad368 - contig02859**

Ad 368:      3' agctaGCCGACTCCCGCTGCCTCCGGTa 5'

                     :|||||:||| | | ||||||:

contig02859: 5' tactgTGGCTGGGGGGGTCCGAGGCCGg 3' from 324 to 350 of X12603

Energy:  -38.11 kCal/Mol

**Ad368 - contig02858**

Ad368:       3' atGCGTGCTTTGGGGCTGGGTCTTAGTCCAg 5'

                  :| |:| ||: |:|||: | || |||||

contig02858: 5' tcTGAATGCAATACTGACTAATAAACAGGTg 3' from 675 to 705 of X12603

Energy:  -20.49 kCal/Mol

**Ad1078 - contig 01024**

Ad1078:      3' atgggtccaGCCTGCTGGCTAAACGTGca 5'

                         | |::| | | |||||||

contig01024: 5' agtaataaaCAGGTGCCAGCTTTGCACtc 3' from 2182 to 2208 of U01027

Energy:  -19.26 kCal/Mol

**Ad1078 - contig00975**

Ad1078:      3' atgggtccaGCCTGCTGGCTAAACGTGca 5'

                         | |::| | | |||||||

contig00975: 5' agtaataaaCAGGTGCCAGCTTTGCACt- 3' from 3016 to 3043 of U01026

Energy:  -18.62 kCal/Mol

**Ad1078 - contig00986**

Ad1078:      3' atgggtccaGCCTGCTGGCTAAACGTGca 5'

                         | |::| | | |||||||

contig00986: 5' agtaataaaCAGGTGCCAGCTTTGCACtc 3' from 36 to 64 of AF269131

Energy:  -19.26 kCal/Mol

**Ad1078 - contig00974**

```
Ad1078:      3' acGATCCGCGGCCGGCTCCGc 5'
                  ||:|| |  |||||||||
contig00974: 5' ggCTGGGGG--GGCCGAGGCc 3' from 1704 to 1722 of U01026
```

Energy:  -31.58 kCal/Mol

**Ad2166 - contig02859**

```
Ad2166:      3' cagCGCAATGGCGTGACCTGCGGAGc 5'
                   | || :| |||  | |:|||||
contig02859: 5' acaGGGTCGCGGCA--GAATGCCTCa 3' from 511 to 534 of X12603
```

Energy:  -22.99 kCal/Mol

**Ad2166 - contig02858**

```
Ad2166:      3' cagCGCAATGGCGTGACCTGCGGAGc 5'
                   | || :| |||  | |:|||||
contig02858: 5' acaGGGTCGCGGCA--GAATGCCTCa 3' from 511 to 534 of X12603
```

Energy:  -22.99 kCal/Mol

**Ad2166 - contig01024**

```
Ad2166:      3' cagCGCAATGGCGTGACCTGCGGAGc 5'
                   | || :| |||  | |:|||||
contig01024: 5' acaGGGTCGCGGCA--GAATGCCTCa 3' from 2008 to 2031 of U01027
```

Energy:  -22.99 kCal/Mol

**Ad2166 - contig01004**

```
Ad2166:      3' atggtCCTTAGGATTGG----CGATCTGGTATACCCt 5'
                     ||||  ||::||    || | |||| |||||
contig01004: 5' gttacGGAAAACTGGCCAAGTGCAACACCAAATGGGa 3' from 1632 to 1668
of U01027
```

Energy:  -29.48 kCal/Mol

**New4393 - contig03089**

New4393: 3' cgatacgaTTGGTGA---TATGG--TGGTTGcg 5'

||:|||| ||:|| ||||||

contig03089: 5' gtcccagaAATCACTTCCATGCCTAACCAACaa 3' from 1433 to 1465 of GQ451440

Energy: -19.42 kCal/Mol

**New4393 - contig03090**

New4393: 3' cgatacgaTTGGTGA---TATGG--TGGTTGcg 5'

||:|||| ||:|| ||||||

contig03090: 5' gtcccagaAATCACTTCCATGCCTAACCAACaa 3' from 2367 to 2399 of D28871

Energy: -19.42 kCal/Mol

**New4393 - contig03078**

New4393: 3' tgtcgtgggccataagggtccgccAGAGGGTa 5'

|||||||

contig03078: 5' ----------aagtcaatgcttccTCTCCCAc 3' from 561 to 582 of HM443642

Energy: -23.29 kCal/Mol

**New2578 - contig03130**

New2578: 3' tgTC-GTGGGCCA-TAAGGGTCCGCCA-GAGGGTAGg 5'

|| |||| | | ||| :|||| || :|| ||||

contig03130: 5' gaAGCCACCGGCTGATTAGTAGGCTGTATTCACATCa 3' from 1212 to 1248 of U01234

Energy: -32.21 kCal/Mol

**New2578- contig03228**

New2578: 3' tgtcGTG-GGCCATAAGGGTC--CG-CCA-GAGGGTAGg 5'

||: |:|| |||| || |: | | ||:|||||

contig03228: 5' atttCATGCTGGCTTTCCAAGATGTAGCTGCTTCCATCa 3' from 1806 to 1845 of GQ451441

Energy: -32.29 kCal/Mol

**New2578 - contig03079**

```
New2578:     3' tgTCGTGGGCCA--TAAGGGTCCGCCA-GAGGGTAGg 5'

                  |||    |||  ||| ::|||| ||  || ||||

contig03079: 5' gaAGCCAGAGGTTGATTATTAGGCTGTAGTCACATCa 3' from 1502 to 1538
of GQ451440
```

Energy:  -26.47 kCal/Mol

**New2578 - contig03128**

```
New2578:     3' agGCGCGGTC--AAGATTC-A-GTCGACGa 5'

                  | :||::|  ||| ||| | :||||||

contig03128: 5' ttCATGCTGGCTTTCCAAGATGTAGCTGCt 3' from 440 to 469 of
GQ451443
```

Energy:  -24.25 kCal/Mol
